# Understanding the Electronic Structure and Diels–Alder Reactivity of Nitrilium-Type *N*‑Hetarynes

**DOI:** 10.1021/acs.joc.6c00572

**Published:** 2026-06-23

**Authors:** Daniel González-Pinardo, Israel Fernández

**Affiliations:** Departamento de Química Orgánica I and Centro de Innovación en Química Avanzada (ORFEO−CINQA), Facultad de Ciencias Químicas, Universidad Complutense de Madrid, Madrid 28040, Spain

## Abstract

The electronic structure and reactivity of the recently reported nitrilium-type *N*-hetarynes were investigated using computational methods. To this end, the diradical character, aromaticity, and Diels–Alder reactivity of these species have been compared with those of their well-established o-benzyne analogues. By means of the activation strain model of reactivity, in combination with the energy decomposition analysis approach, it is found that a significant reduction of the Pauli repulsion between the reactants is behind the enhanced reactivity of the heterocyclic aryne. Furthermore, this study also examines how substituents directly attached to the boron atom in 1,2-azaborine-derived 1,6-BN-arynes, as well as the nature of the group-13 element adjacent to the nitrilium fragment, influence the reactivity of these novel species.

## Graphical abstract

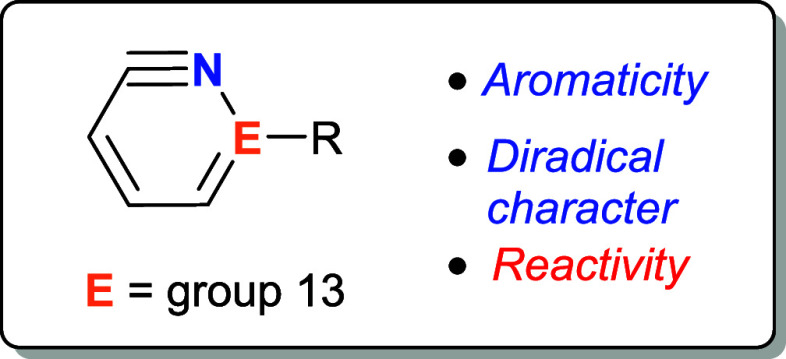

## Introduction

Arynes constitute a family of transient species featuring a highly strained triple bond embedded in an aromatic framework. Since the proposal of the parent benzyne as an intermediate by Bachmann and Clarke in 1927 ^1^ and later by Wittig in 1942,^2^ these species have long fascinated chemists, not only because of their unique structural and electronic properties, but especially due to their countless applications in synthesis.^3,4^ Indeed, arynes typically function as polarized two-carbon synthons, enabling the 1,2-functionalization of arenes, and they have widely been used for the preparation of a number of target molecules, including natural products^5^ and systems relevant to materials science.^4^

Although the family of arynes has recently expanded to include the so-called hetarynes (i.e., heterocyclic arynes) such as pyridynes and indolynes (Scheme 1),^6,7^ which serve as versatile building blocks for the construction of complex systems, the chemistry of the corresponding CN-type (nitrilium) *N*-hetarynes is comparatively underdeveloped. This is mainly because of the difficulties associated with the preparation of these species. For instance, whereas the parent 1,2-pyridyne has only been observed in the gas phase,^8^ the related 1,2-indolynes or 1,2-pyrrolynes have so far remained elusive. Fortunately, based on the previous isolation of the 1,2-azaborine-derived 1,2-BN-aryne,^9^ Liu, Bettinger, and coworkers very recently achieved the first successful solution-phase synthesis of a CN-type hetaryne (**1**) through a base-promoted HBr elimination from the precursor C6-bromo-B-mesityl-1,2-azaborine.^10^ Interestingly, this species was found to be highly reactive, participating in different cycloaddition reactions (including [4 + 2], [3 + 2], or [2 + 2] processes) as well as electrophilic aromatic substitutions with electron-rich arenes (Scheme 1).

**Scheme 1:**
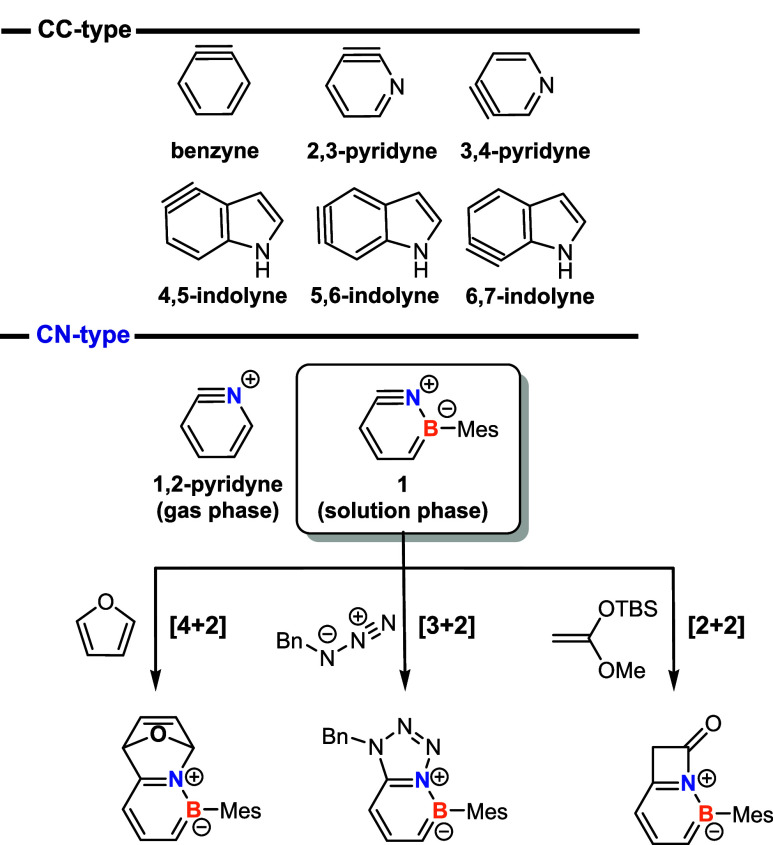
Selected Examples of CC and CN-Type Arynes and Hetarynes

Despite the similarities in reactivity between CN-hetaryne **1** and CC-arynes—both undergo cycloaddition and nucleophilic reactions—very little is known about the influence of the particular electronic structure of **1** on its reactivity. This prompted us to carry out a comprehensive comparative study of the factors controlling the reactivity of **1** in comparison to the well-established reactivity and electronic structure of its benzyne-type analogue. To this end, we will focus on the [4 + 2] cycloaddition reaction with furan and apply the combination of the activation strain model (ASM)^11^ and energy decomposition analysis (EDA)^12^ methods, which have proven helpful for our current understanding of fundamental processes in chemistry,^13^ and particularly, cycloaddition reactions.^14^ Furthermore, we will also analyze additional factors influencing the reactivity of the hetaryne, such as the impact of the substituents directly attached to the boron atom and the nature of the group 13 element bonded to the nitrogen atom.

## Results and Discussion

### Electronic Structure and Aromaticity

Previous computational studies have shown that the triple bond of the parent o-benzyne exhibits a non-negligible biradical character.^15^ Our calculations further support this conclusion: at the CASSCF­[2,2]/def2-TZVPP//ωB97X-D/def2-TZVPP level, the computed Natural Orbital Occupation of the lowest unoccupied natural orbital (n_LUNO_), which provides a reliable measure of the diradical character,^16^ for **1-C**_**Mes**_, the isolectronic CC-aryne analogue of hetaryne **1**, is n_LUNO_ = 0.18e. At variance, the corresponding value for the CN-hetaryne **1**, calculated at the same level of theory, is n_LUNO_ = 0.07e, which clearly indicates that this species is best described as a closed-shell singlet with negligible biradical character.

In addition, it has also been reported that the bonding situation of o-benzyne is best represented by a triple-bonded (i.e., strained alkyne) valence structure.^15b^ By analogy, hetaryne **1** can be viewed as a resonance hybrid between two main forms, namely the nitrilium **1-A**, featuring the strained C≡N triple bond, and the cumulene-like (i.e., ketenimine) resonance form **1-B** (Scheme 2). Our Natural Resonance Theory (NRT)^17^ analysis of **1** suggests that both resonance forms contribute comparably to its electronic structure, with the nitrilium form being slightly favored.

**Scheme 2:**
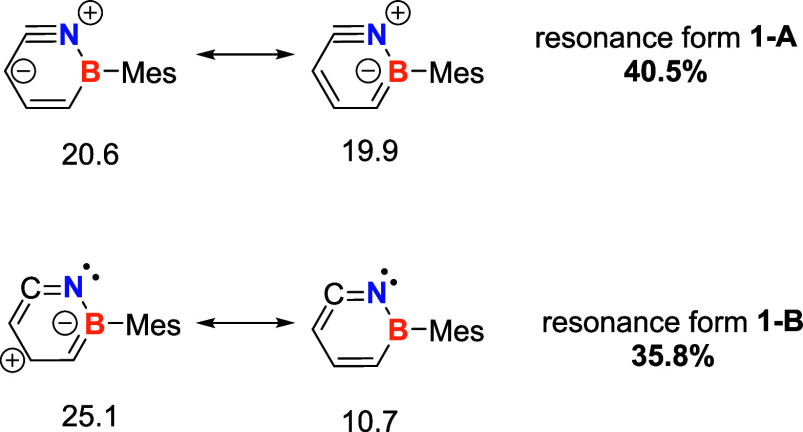
Natural Resonance Theory (NRT) Analysis of **1** Showing the Contribution of Each Resonance From (in percentage)^a^

a All data have been computed at the wB97X-D/def2-TZVPP level.

Finally, o-benzyne has also been repeatedly reported as an aromatic molecule according to geometric, energetic, and magnetic criteria.^18^ For comparison, we were also curious to explore the aromatic nature of hetaryne **1**. We first applied the Anisotropy of the Induced Current Density (ACID)^19^ method, which reveals the presence of diatropic (clockwise vectors) ring currents in both **1** and its benzyne counterpart **1-C**_**Mes**_ (see Figure 1a), therefore confirming the (magnetic) aromatic nature of both arynes. In addition, the computed Nucleus-Independent Chemical Shift (NICS)^20^ values at the (3,+1) ring critical point of the electron density;^21^ specifically, the reliable out-of-plane component computed 1 Å above the ring center (NICS(1)_*zz*_),^22^ indicate that hetaryne **1** is less aromatic than its CC-analogue **1-C**_**Mes**_ (NICS(1)_*zz*_ = −24.6 ppm vs −32.8 ppm, respectively, see Figure 1a). This is also supported by the computed Aromatic Stabilization Energy (ASE) values, calculated using the Schleyer/Pühlhofer isomerization method,^23^ which further indicate that the ASE is lower for **1** than for **1-C**_**Mes**_ (ASE = 21.7 kcal/mol vs 32.8 kcal/mol, respectively, see Figure 1b). Therefore, our calculations clearly suggest that although **1** can be considered as an aromatic species, its aromaticity is reduced as compared to its benzyne analogue.

**Figure 1:**
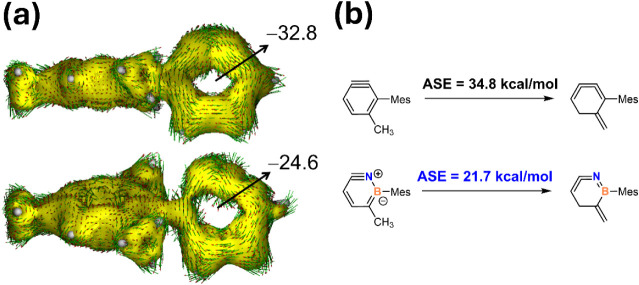
(a) Computed ACID plots (isosurface value of 0.03 au) and NICS(1)*_zz_* values (in ppm) for **1-C_Mes_** and **1**. (b) ASE values computed by means of the isomerization method.

### Reactivity of 1-C_Mes_ and 1-B_Mes_

Once the electronic structure of the hetaryne **1** was studied, we then explored the reactivity of this species in comparison with its o-benzyne analogue (**1-C**_**Mes**_). To this end, we focused on the Diels–Alder cycloaddition reaction involving furan as the diene. As shown in the computed reaction profiles depicted in Figure 2, both cycloaddition reactions start with the endergonic formation of an initial van der Waals complex **RC/RC-C**_**Mes**_ (whose formation becomes exothermic when entropic effects are not considered), which is then transformed into the corresponding cycloadduct (**2** and **2-C**_**Mes**_) in an highly exergonic process (ΔG_R_ = −30.2 and −49.7 kcal/mol, respectively). The difference in the computed exergonicity can be ascribed first to the weaker C–N bond formed in the process involving **1** as compared to the stronger C–C bond formed in the analogous reaction involving **1-C**_**Mes**_.^24^ In addition, there exists a more substantial strain release in the carbocyclic framework (C–C≡C angle of 126.9° in **1-C**_**Mes**_ vs 146.3° for the C–C≡N angle in **1**), which also contributes to the higher exergonicity of the corresponding process. As shown in Figure 2, the cycloadditions proceed in both cases in a concerted manner through the corresponding highly asynchronous six-membered transition states (**TS** and **TS-C**_**Mes**_). Interestingly, the asynchronicity in these saddle points is inverted, i.e., whereas the shortest C···C bond-forming distance in **TS-C**_**Mes**_ involves the C2 carbon atom of the benzyne (proximal to the mesityl group), the shortest bond-forming distance in **TS** involves the C1 of the hetaryne. This directly results from the remarkable polarization induced by the (nitrilium) nitrogen atom in **1**, as reflected in the highly positive charge at C1 (computed NBO charge of +0.56), which favors the interaction with the diene at this position. In addition, this is also consistent with Houk-Garg’s aryne distortion model,^25^ as the internal C–C1≡N angle is significantly higher (i.e., more distorted) than the C1≡N–B angle (146.3° vs 115.4°, respectively). In contrast, the internal angles in **1-C**_**Mes**_ are rather similar (126.9° and 128.8°), suggesting that the asynchronicity of this reaction mainly derives from the electronic effects induced by the mesityl group. This is supported by a comparison with the parent hetaryne system (R = H, **1-C**_**H**_), whose corresponding transition state is completely synchronous (C···C bond-forming distances of 2.46 Å). Finally, from the low computed barriers, both processes are also kinetically feasible at room temperature, which agrees with the observed high reactivity of these arynes. Despite that, the process involving the hetaryne proceeds with a lower barrier than that involving the o-benzyne analogue (ΔΔG^‡^ = 1.6 kcal/mol), thus indicating that **1** exhibits an enhanced reactivity as compared to **1-C**_**Mes**_.

**Figure 2:**
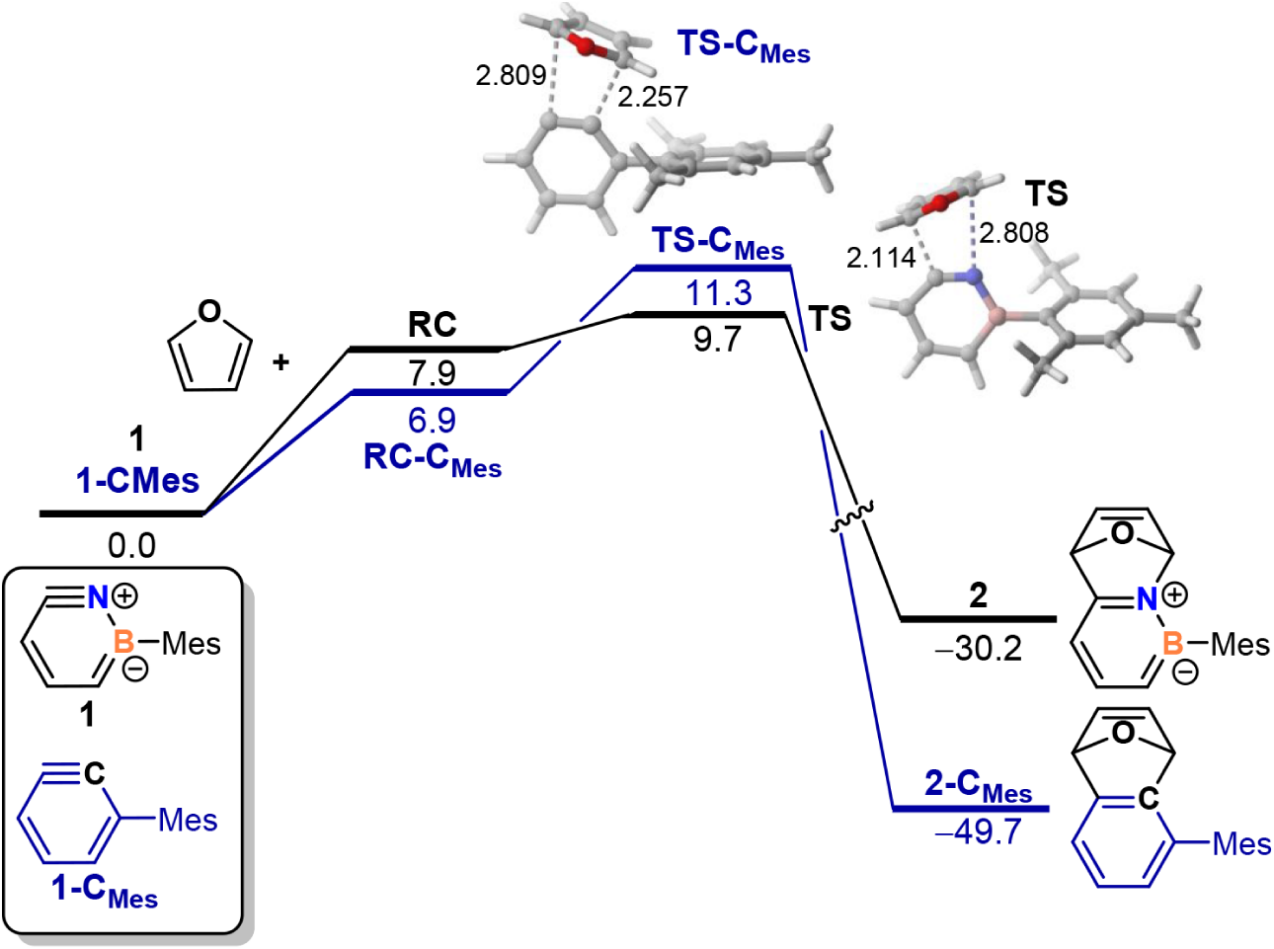
Computed reaction profiles for the [4 + 2] cycloaddition between furan and **1** (black) and **1-C_Mes_** (blue). Relative free energies (ΔG, at 298 K) and bond distances are given in kcal/mol and angstroms, respectively. All data were computed at the DLPNO–CCSD­(T)/def2-TZVPP//ωB97X-D/def2-TZVPP level.

To understand, in a quantitative manner, the factors behind the higher reactivity of the CN-hetaryne **1** with respect to its CC-counterpart, the Activation Strain Model (ASM) of reactivity^11^ was applied next. This approach decomposes the electronic energy (ΔE) into two terms, namely the strain (ΔE_strain_) that results from the distortion of the individual reactants and the interaction (ΔE_int_) between the deformed reactants along the reaction coordinate, defined in this case by the shorter C···C bond-forming distance in the cycloaddition with furan.^26,27^ Figure 3a shows the corresponding Activation Strain Diagrams (ASDs) for both processes, from the initial stages of the transformations up to the corresponding transition states. From the data in Figure 3a, it becomes clear that the lower barrier computed for the cycloaddition reaction involving **1** results from both a less destabilizing strain energy, which comes from its less strained structure (see above), and a stronger interaction between the deformed reactants along the entire reaction coordinate. For instance, at the same consistent C···C bond-forming distance of 2.25 Å,^28^ the differences in the strain energy, ΔΔE_strain_ = 2.0 kcal/mol, and the interaction energy, ΔΔE_int_ = 0.7 kcal/mol, both favor the **1** + furan cycloaddition reaction.

**Figure 3:**
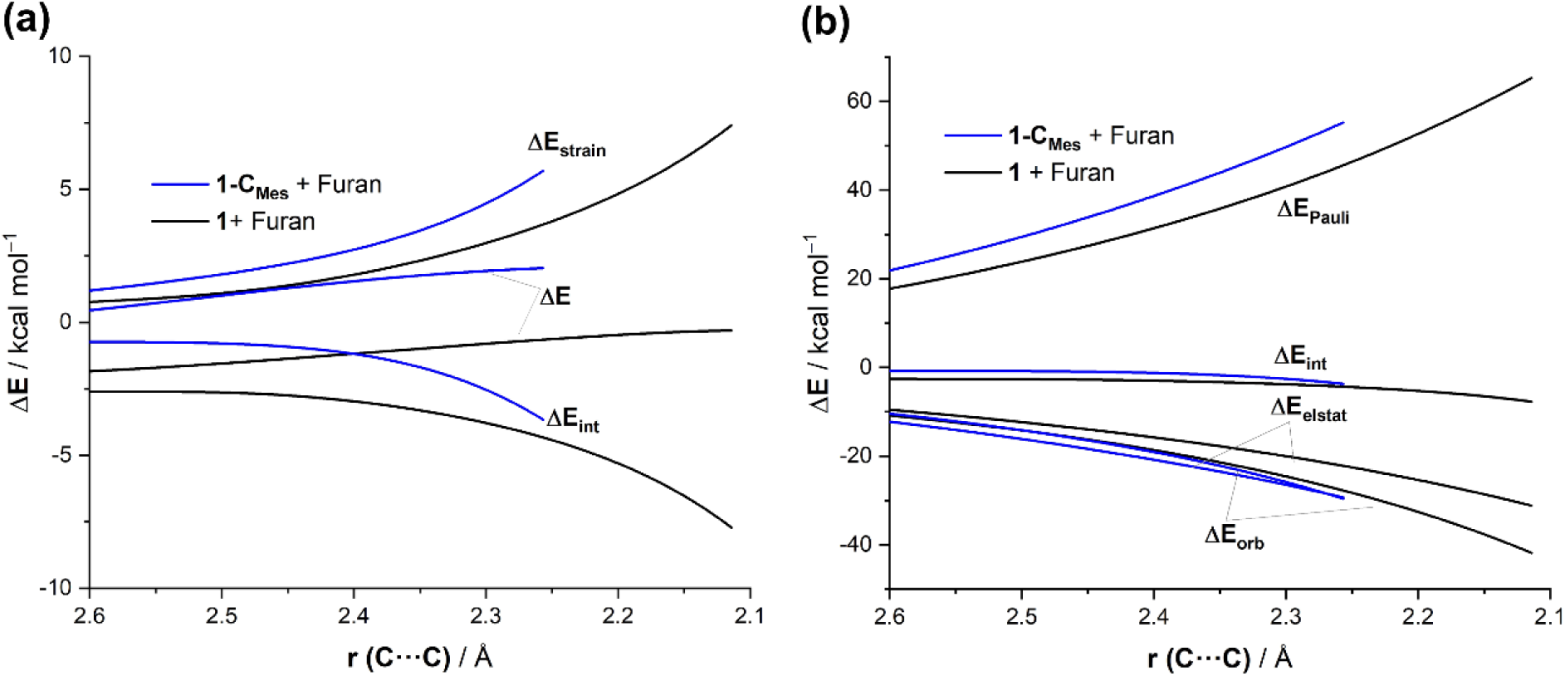
Comparative activation strain diagrams (a) and energy decomposition analysis (b) for the [4 + 2] cycloaddition reaction between furan and **1** (black) or **1-C_Mes_** (blue) and projected onto the forming C···C shortest bond distances. All data were computed at the ZORA-ωB97X-D/TZ2P//ωB97X-D/def2-TZVPP level.

We then applied the Energy Decomposition Analysis (EDA) method^12^ to understand why the interaction energy between the deformed reactants becomes stronger (*i.e*., more stabilizing) for the process involving the hetaryne **1**. Our canonical EDA involves decomposing the interaction ΔE_int_ between the reactants into the following energy terms: the classical electrostatic interaction (ΔE_elstat_), the Pauli repulsion (ΔE_Pauli_) arising from the repulsion between occupied orbitals of both deformed reactants, and the orbital interaction (ΔE_orb_) that accounts for charge transfer and polarization. As graphically shown in Figure 3b, the stronger interaction computed for the cycloaddition involving **1** does not derive from the orbital term (ΔE_orb_) or the electrostatic interactions (ΔE_elstat_), which are actually more stabilizing for the o-benzyne transformation. At variance, the Pauli repulsion (ΔE_Pauli_) is clearly less destabilizing for the **1** + furan cycloaddition and compensates for the attractive energy terms, resulting in a stronger interaction energy along the entire reaction coordinate.

The origin of the less destabilizing Pauli repulsion for the transformation involving **1** can be found by performing a Kohn–Sham molecular orbital analysis. The main contribution to the total Pauli repulsion comes from the 4-electron/two-orbital interaction between the doubly occupied in-phase π molecular orbital of furan (HOMO–1) and the π-molecular orbital of the aryne located at the C≡E bond (see Figure 4). The corresponding orbital overlap (*S*) between these molecular orbitals, computed at the same consistent C···C bond-forming distance of 2.25 Å, is clearly much smaller for the process involving **1** (*S* = 0.025) than that computed for the analogous reaction involving **1-C**_**Mes**_ (*S* = 0.065). This, again is a consequence of the different strained nature of the triple bond and, to a greater extent, of the polarization exerted by the N–B moiety, which significantly reduces the population of the C≡N bond in comparison to the analogous C≡C bond. Therefore, it can be concluded that the higher reactivity of hetaryne **1** results mainly from a smaller overlap between the key π-molecular orbitals in both reactants, which translates into a stronger interaction between the reactants along the reaction coordinate and ultimately into a lower activation barrier.

**Figure 4:**
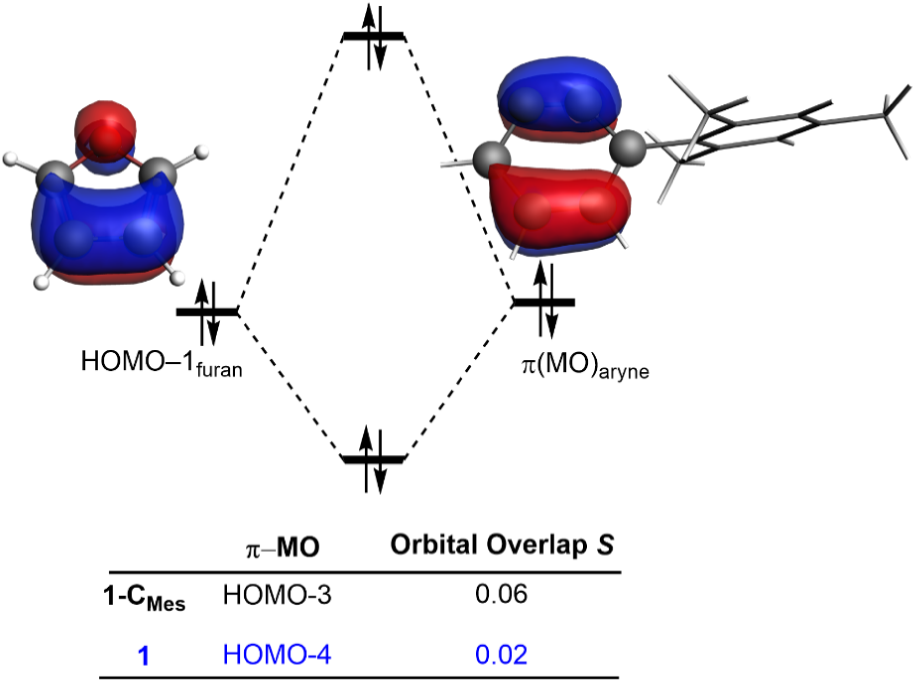
Schematic orbital-interaction diagram of the most significant occupied orbital overlap of the cycloaddition reaction between furan and **1** and **1-C_Mes_**. Data were computed at the ZORA-ωB97X-D/TZ2P//ωB97X-D/def2-TZVPP level at a consistent C···C bond-forming distance of 2.25 Å.

### Tuning the Reactivity of the Hetaryne

#### Influence of the Boron Fragment

Results above strongly suggest that the reactivity of the hetaryne **1** can be tuned by simply controlling the electronic properties of the adjacent boron fragment. We hypothesized that if the electron-withdrawing ability of the boron moiety increases, then the reduction in the population of the C≡N bond would be more significant, and this should translate into a further reduction of the activation barrier. To test this hypothesis, we compared the cycloaddition reactions of furan with a series of CN-type heterarynes analogous to **1**, in which the substituents directly attached to the boron atom have varying donor/acceptor abilities. From the data in the computed reaction profiles presented in Figure 5, it is confirmed that the presence of electron-withdrawing groups (**1-B**_**A**_, **1-B**_**AA**_) leads to a lower barrier than the parent hetaryne **1** (ΔΔG^‡^ ≈ −2 kcal/mol for **1-B**_**AA**_, B­(C_6_F_5_)), whereas donor groups provoke the opposite effect and increase the barrier of the cycloaddition (ΔΔG^‡^ ≈ 3 kcal/mol for **1-B**_**DD**_, B­(NMe_2_)). Interestingly, the donicity of the substituent also has an impact on the nature of the corresponding (asynchronous) transition state. As shown in Figure 5, the key shorter C···C bond-forming distance becomes shorter and shorter as the donicity of the substituent increases, which indicates that electron-withdrawing groups lead to earlier transition states, whereas the respective transition states are reached later when electron-donor groups are attached to the boron atom.

**Figure 5:**
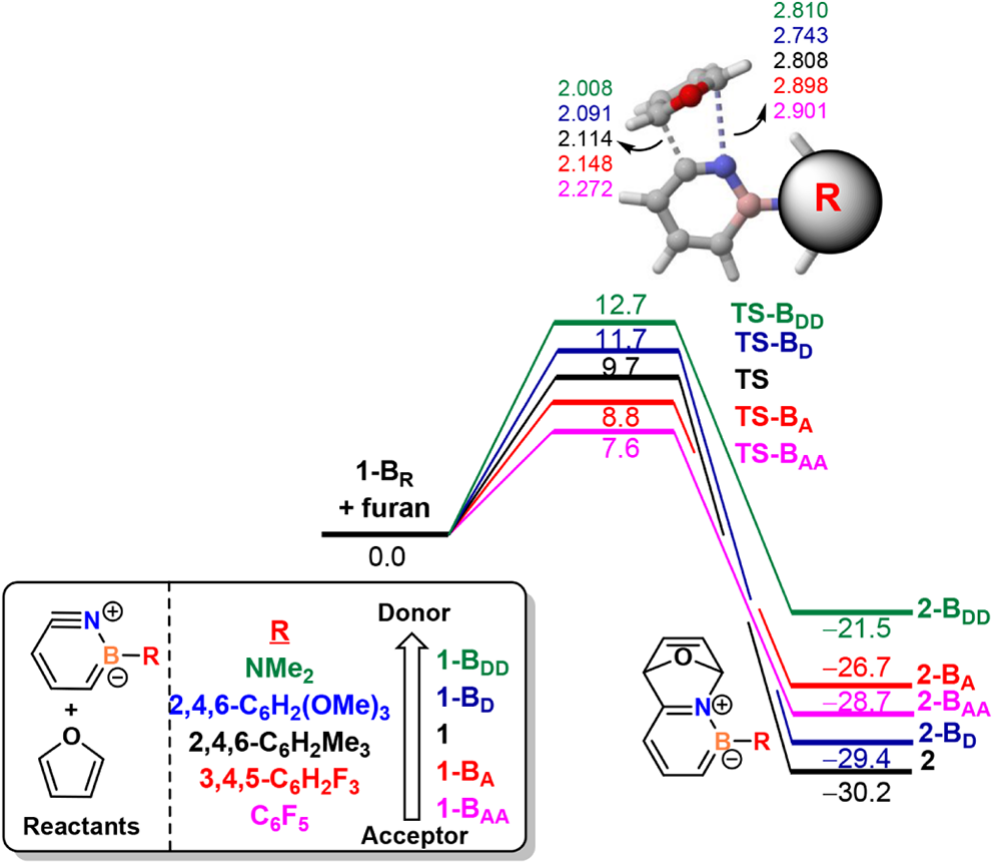
Computed reaction profiles for the [4 + 2] cycloaddition between furan and **1-B_R_** with different substituents attached to the boron atom (see inset). Relative free energies (ΔG, at 298 K) and bond distances are given in kcal/mol and angstroms, respectively. All data were computed at the DLPNO–CCSD­(T)/def2-TZVPP//ωB97X-D/def2-TZVPP level.

The combination of the ASM-EDA method was also applied to understand the reactivity trend computed for these cycloadditions involving B-substituted hetarynes. To this end, we compared the cycloadditions involving **1-B**_**A**_ (R = 3, 4, 5-C_6_H_2_F_3_) and **1-B**_**DD**_ (R = NMe_2_) as representative reactions having electron-acceptor and electron-donor groups, respectively. The corresponding ASDs, once again computed from the initial stages of the transformation up to the corresponding transition states (Figure 6a), confirm that the process involving **1-B**_**A**_ benefits from a much stronger interaction between the deformed reactants along the entire process. This compensates for the less destabilizing strain computed for the process involving **1-B**_**DD**_, which again mainly results from its less strained structure (C–C≡N angle of 148.5° in **1-B**_**DD**_ vs 147.3° for the C–C≡N angle in **1-B**_**A**_). According to the EDA (Figure 6b), the stronger interaction computed for the processes involving the hetaryne featuring the electron-withdrawing group, results, as expected, from a slight reduction in the Pauli repulsion during the entire reaction coordinate. This once again originates from a smaller overlap between the key π-molecular orbitals in both reactants: at the same consistent C···C bond-forming distance of 2.15 Å, *S* < HOMO–1_furan_|π (C≡N)_aryne_) is 0.025 for the process involving **1-B**_**A**_, whereas a higher value of *S* = 0.031 was computed for the analogous reaction involving **1-B**_**DD**_. Despite that, the main factor leading to the stronger interaction is the orbital term (ΔE_orb_), which as shown in Figure 6b, becomes stronger for the **1-B**_**A**_ + furan cycloaddition along the entire reaction coordinate.

**Figure 6:**
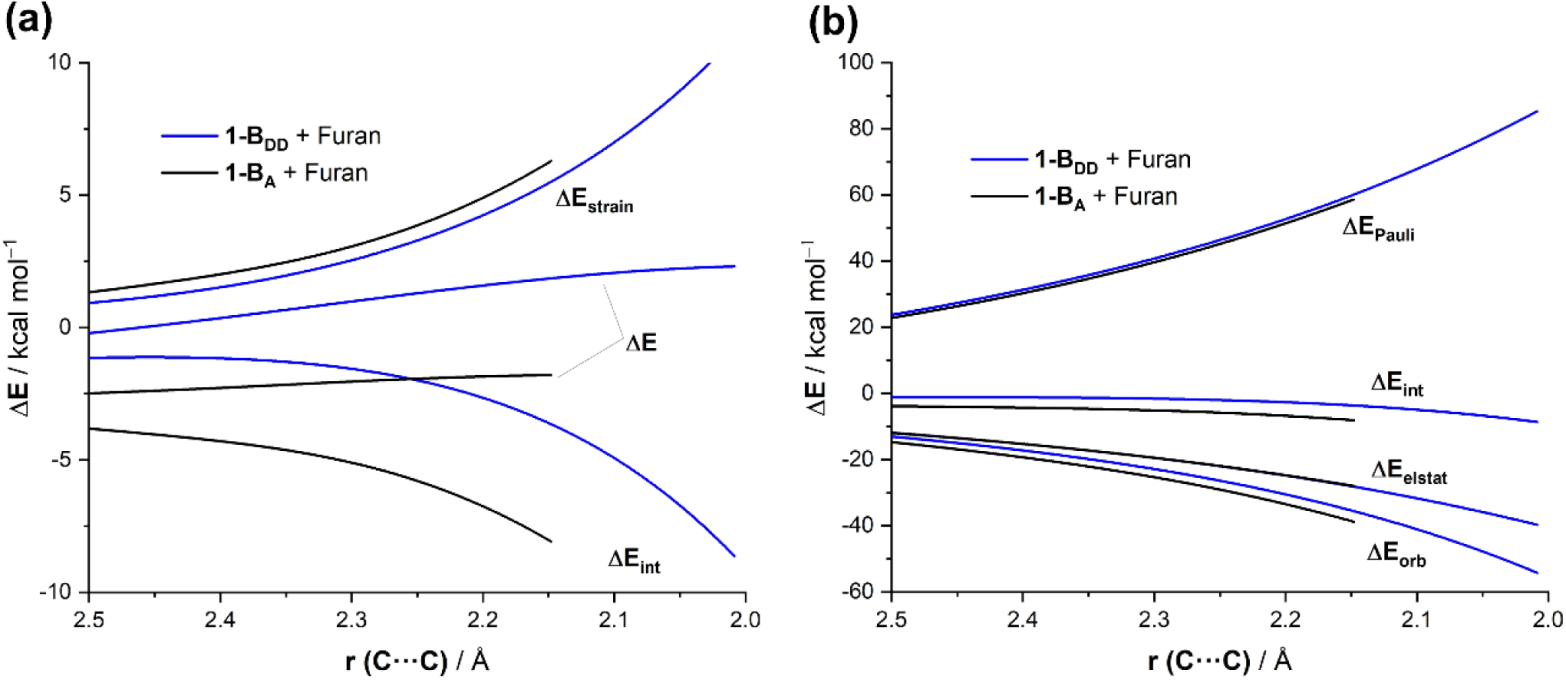
Comparative activation strain diagrams (a) and energy decomposition analysis (b) for the [4 + 2] cycloaddition reaction between furan and **1-B_DD_** (blue) and **1-B_A_** (black), projected onto the shortest C···C bond-forming distances. All data were computed at the ZORA-ωB97X-D/TZ2P//ωB97X-D/def2-TZVPP level.

We next applied the Natural Orbital for Chemical Valence (NOCV)^29^ extension of the EDA to gain more insight into the reasons behind the observed enhanced orbital interactions computed for the cycloaddition involving **1-B**_**A**_. This method allows us not only to visualize but also to quantify the main molecular orbital interactions in the process. According to the EDA-NOCV, the transformation is dominated by the donation of electron density from the doubly occupied π­(HOMO) molecular orbital of furan to the vacant π* molecular orbital of the hetaryne (mainly located at the C≡N moiety, see Figure 7). This confirms the normal electronic demand nature of the cycloaddition, regardless of the nature of the substituent attached to the boron atom. Interestingly, the stabilization energy associated with this π­(diene) → π*­(dienophile) interaction is higher for the reaction involving **1-B**_**A**_ than that for the analogous process involving **1-B**_**DD**_ (see energy values in Figure 7 computed at the same consistent C···C bond-forming distance of 2.15 Å). This translates into the computed higher total ΔE_orb_ and stronger total interaction between the deformed reactants, and ultimately into the lower barrier computed for the process involving the electron-withdrawing group.

**Figure 7:**
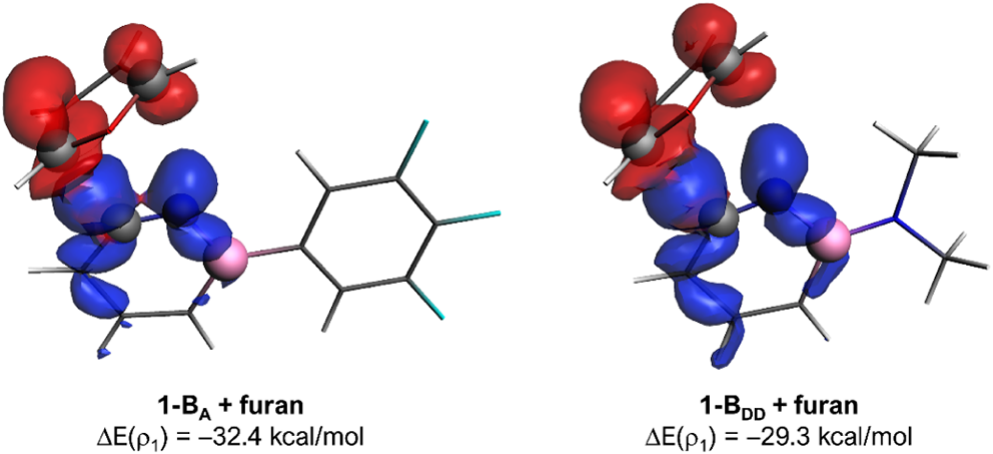
Contour plots of the main NOCV deformation densities ρ (isosurface value of 0.001 au) and associated energies ΔE­(ρ) for the [4 + 2] cycloaddition reaction between furan and **1** (left) and **1-Al** (right), computed at the same consistent C···C bond-forming distance of 2.15 Å. The electronic charge flows from red to blue. All data were computed at the ZORA-ωB97X-D/TZ2P//ωB97X-D/def2-TZVPP level.

#### Influence of the Group 13 (E = B, Al, Ga, In) Atom

For completeness, we were also curious to explore the influence of the group 13 atom directly attached to the nitrogen atom of the hetaryne on its reactivity. To this end, we compared the same [4 + 2] cycloaddition reaction involving furan and **1** with the analogous transformations where the boron atom was replaced by its heavier counterparts (**1-Al**, **1-Ga,** and **1-In**). As expected, the computed reaction profiles (Figure 8) confirm that, in all cases, the cycloadditions take place in a concerted manner through the corresponding asynchronous transition states **TS-E,** which lead to the formation of the respective cycloadducts **2-E**. From the data in Figure 8, a clear reactivity trend emerges: the process becomes less and less exergonic, and the activation barrier becomes higher and higher when going down in the group 13. As a consequence, it is not surprising that a clear linear relationship between the two computed energies (ΔG^‡^ and ΔG_R_) was found (ΔG^‡^ = 28.99 + 0.65 ΔG_R_, correlation coefficient, R^2^ = 0.995). Interestingly, the slope of this linear correlation is close to 0.5, which suggests that the considered Diels–Alder cycloaddition reactions follow the empirical relationship ΔE^‡^=ΔE_0_^‡^ + ΔE_R_/2, given by Brønsted, Dimroth, Marcus, and Bell–Evans–Polanyi (also known as the Bema Hapothle relationship).^30^ Thus, the data in Figure 8 allow us to predict that, different from the B-system **1**, their heavier analogues, and particularly the In derivative **1-In**, are much less reactive, and therefore, might be isolated or detected experimentally.

**Figure 8:**
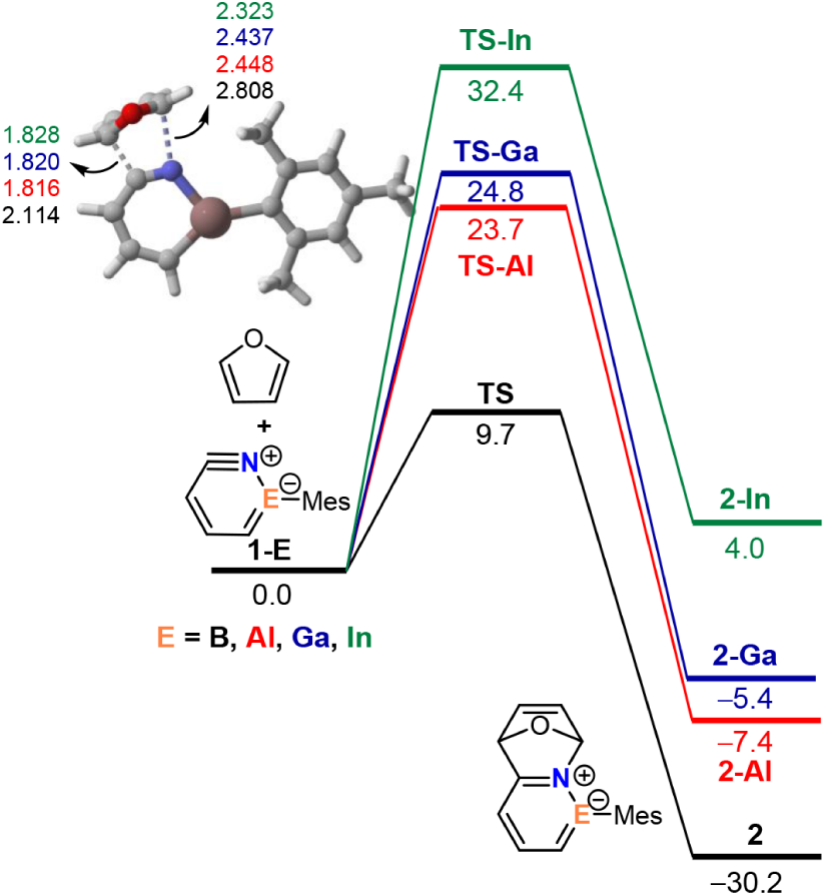
Computed reaction profiles for the [4 + 2] cycloaddition between furan and **1-E** (E = B, Al, Ga, In), Relative free energies (ΔG, at 298 K) and bond distances are given in kcal/mol and angstroms, respectively. All data were computed at the DLPNO–CCSD­(T)/def2-TZVPP//ωB97X-D/def2-TZVPP level.

The ASM-EDA method was finally applied to understand the factors leading to the enhanced reactivity of hetaryne **1** in comparison with its heavier counterparts. To this end, we compared the cycloaddition reaction involving **1** and the analogous process involving **1-Al**. The corresponding ASDs, once again from the beginning of the reactions up to the respective transition states and projected onto the shortest C···C bond-forming distance (Figure 9a), clearly indicate that the higher reactivity of **1** is not at all due to the strain energy, which is nearly identical for both processes. At variance, the **1** + furan reaction benefits from a stronger interaction energy between the deformed reactants along the entire transformation. Therefore, it can be concluded that the higher reactivity of **1** in comparison with its heavier analogues derives exclusively from the stronger interaction between the deformed reactants. According to the EDA (Figure 9b) and similar to the cycloadditions involving the B-substituted systems, this stronger interaction results from both a less destabilizing Pauli repulsion and stronger orbital interactions. The former derives from a smaller orbital overlap between the doubly occupied key π-molecular orbitals in both reactants (*S* = 0.027 vs *S* = 0.032, for **1** and **1-Al**, respectively, computed at the same consistent distance of 2.11 Å), whereas the latter, according to the EDA-NOCV method, results once again from a more stabilizing π­(diene) → π*­(dienophile) interaction for the process involving the boron-hetaryne **1** (see corresponding deformation densities and stabilizing energies in Figure 10). This therefore suggests that the B-Mes group increases the acceptor ability of the π*­(C≡N) molecular orbital of the hetaryne in its cycloaddition with furan in comparison with its heavier E-Mes counterparts.

**Figure 9:**
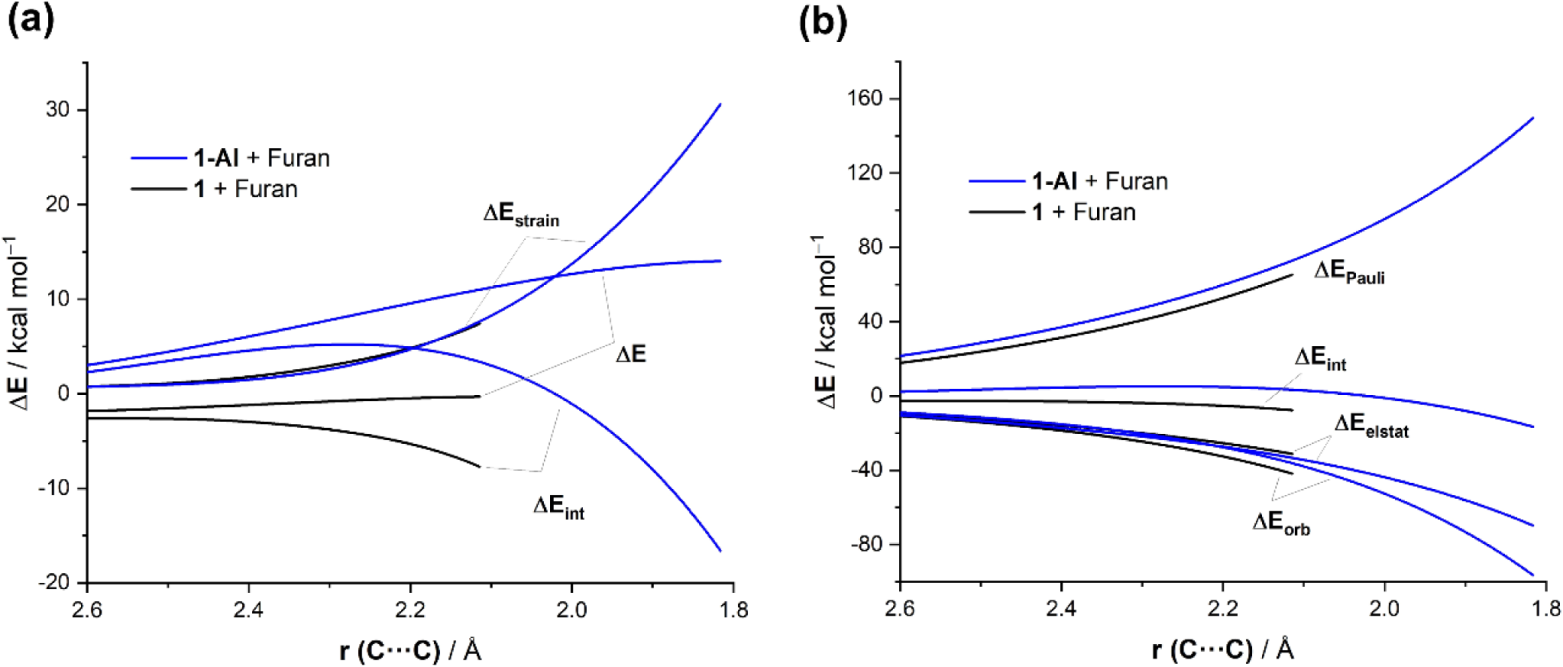
Comparative activation strain diagrams (a) and energy decomposition analysis (b) for the [4 + 2] cycloaddition reaction between furan and **1** (black) and **1-Al** (blue), projected onto the shortest C···C bond-forming distances. All data were computed at the ZORA-ωB97X-D/TZ2P//ωB97X-D/def2-TZVPP level.

**Figure 10:**
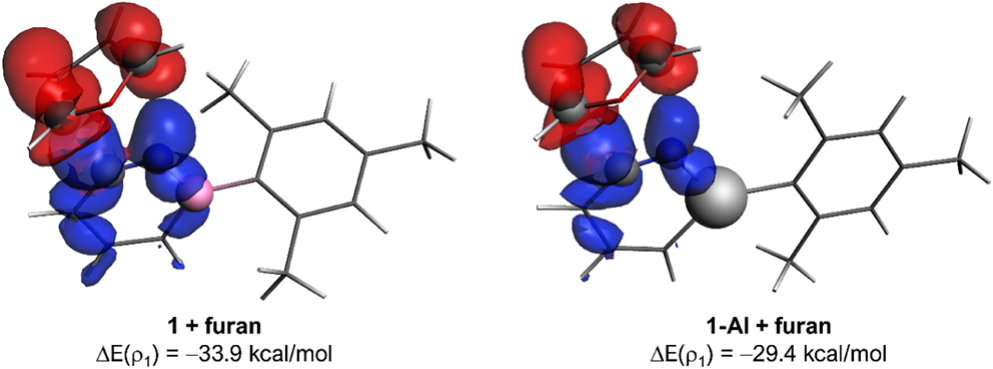
Contour plots of the main NOCV deformation densities ρ (isosurface value of 0.001 au) and associated energies ΔE­(ρ) for the [4 + 2] cycloaddition reaction between furan and **1** (left) and **1-Al** (right) computed at the same consistent C···C bond-forming distance of 2.1 Å. The electronic charge flows from red to blue. All data have been computed at the ZORA-ωB97X-D/TZ2P//ωB97X-D/def2-TZVPP level.

## Conclusions

In this work, the interplay between the electronic structure, aromaticity, and reactivity of nitrilium-type *N*-hetarynes has been investigated using computational methods. Compared with its o-benzyne analogue, the recently reported 1,2-azaborine-derived 1,6-BN-aryne is found to be less aromatic, to possess negligible diradical character, and to be significantly more reactive in its cycloaddition with furan. This enhanced reactivity arises mainly from a significant reduction in the Pauli repulsion between the reactants as a consequence of the strong polarization exerted by the N–B fragment on the reactive C≡N bond. This reactivity can be readily tuned by modifying the donor/acceptor properties of the substituent directly attached to the boron atom: electron-withdrawing groups increase reactivity, whereas donor groups provoke the opposite effect. This is once again due to a reduction of Pauli repulsion in the process, which highlights the crucial role of this factor in the process, and to an enhancement of the π (diene)→ π*­(dienophile) molecular orbital interaction. Finally, the replacement of the boron atom by its heavier group 13 element counterparts leads to a marked decrease in hetaryne reactivity, particularly for E = In. This can be once again ascribed mainly to a significant reduction of the key π­(diene) → π*­(dienophile) molecular orbital interactions.

Overall, this study provides new insights into the chemistry of the recently introduced nitrilium-type *N*-hetarynes, which may aid in the further development of this family of compounds.

## Computational Methods

Geometry optimizations of the molecules were performed without symmetry constraints using the Gaussian16 (RevB.01) suite of programs^31^ at the (gas phase)-ωB97xD^32^/def2-TZVPP^33^ level. Reactants and products were characterized by frequency calculations and were found to have positive definite Hessian matrices. Transition states exhibited only one negative eigenvalue in their diagonalized force constant matrices, and their associated eigenvectors were confirmed to correspond to the motion along the reaction coordinate under consideration using the Intrinsic Reaction Coordinate (IRC) method.^34^ Energy refinements were carried out by means of single-point calculations at the Domain-Based Local Pair-Natural Coupled-Cluster (DLPNO–CCSD­(T), using NormalPNO)^35^ level with the Orca 6.1.1 program^36^ using the def2-TZVPP^32^ basis set on the ωB97xD/def2-TZVPP optimized geometries. This level of theory is denoted as DLPNO–CCSD­(T)/def2-TZVPP//ωB97xD/def2-TZVPP. The computed thermochemistry data were corrected following Grimme’s quasi-harmonic (QHA) model for entropy^37^ with a frequency cutoff value of 100.0 cm^–1^ using the GoodVibes^38^ program at 298.15 K. State Specific Complete Active Space Self-Consistent Field (SS-CASSCF) calculations were performed in ORCA.

The program package ADF^39^ was used for ASM and EDA calculations using the optimized ωB97X-D/def2-TZVPP geometries at the same DFT level, in conjunction with a triple-ς-quality basis set using uncontracted Slater-type orbitals (STOs) augmented by two sets of polarization functions, with a frozen-core approximation for the core electrons.^40^ Auxiliary sets of s, p, d, f, and g STOs were used to fit the molecular densities and to represent the Coulomb and exchange potentials accurately in each SCF cycle.^41^ Scalar relativistic effects were incorporated by applying the zeroth-order regular approximation (ZORA).^42^ This level of theory is denoted ZORA-ωB97X-D/TZ2P//ωB97X-D/def2-TZVPP.

## Supplementary Material



## Data Availability

The data underlying this study are available in the published article and its Supporting Information.

## Notes

Cartesian coordinates (in Å) and energies for all structures discussed in the text (PDF) (PDF)
