# Supplementary material for: Understanding the Electronic Structure and Diels–Alder Reactivity of Nitrilium-Type N‑Hetarynes
Source: J Org Chem. 2026 Jun 23;91(26):8881–9. doi: 10.1021/acs.joc.6c00572 (PMC13339620; doi:10.1021/acs.joc.6c00572)
Supplement: Supplementary file 1 [file jo6c00572_si_001.pdf]

Understanding the Electronic Structure and Diels-Alder Reactivity of Nitrilium-Type *N*-Hetarynes

Daniel González Pinardo[†], and Israel Fernández[†]*

[†]Departamento de Química Orgánica I and Centro de Innovación en Química Avanzada (ORFEO-CINQA), Facultad de Ciencias Químicas, Universidad Complutense de Madrid, 28040-Madrid, Spain.

e-mail: israel@quim.ucm.es

Contents:

1. Figure S1……………………………………………………………………………..S1

2. Cartesian coordinates…..………………………………………….…………..……S2

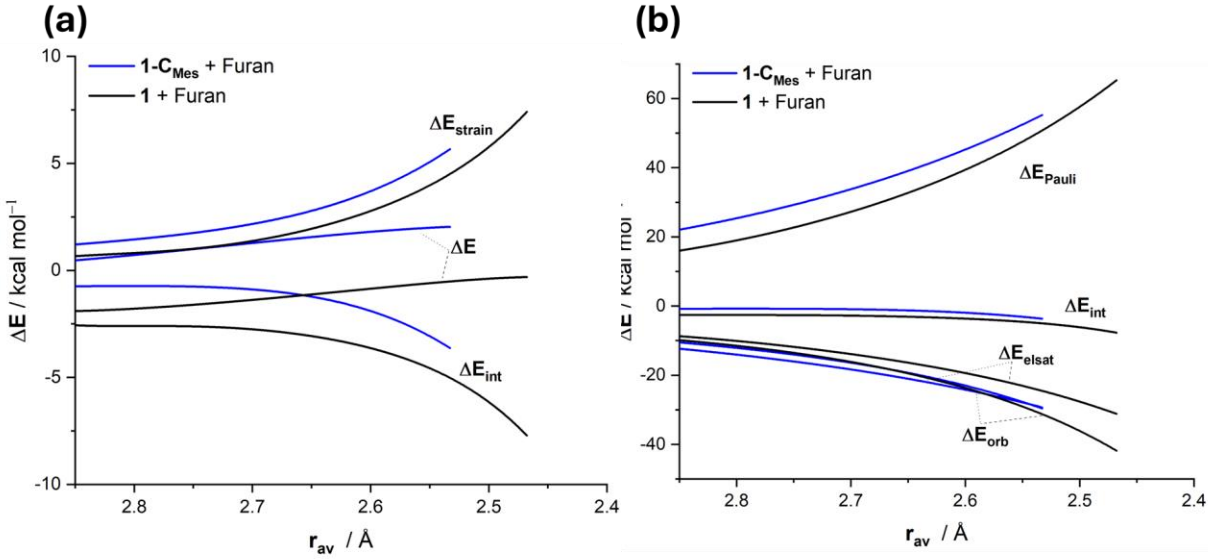

Figure S1. Comparative activation strain diagrams (a) and energy decomposition analysis (b) for the [4+2] cycloaddition reaction between furan and **1** (black) or **1-C$_{Mes}$** (blue) and projected onto the average C···C / C···N (for **1**) C···C (for **1-C$_{Mes}$**) bond-forming distances. All data have been computed at the ZORA-ωB97X-D/TZ2P//ωB97X-D/def2-TZVPP level.

1. Cartesian coordinates of the DFT optimized structures

Cartesian coordinates (in Å) and total energies (in a.u., noncorrected ZVPE included) of all the stationary points discussed in the text. Unless otherwise mentioned, all calculations have been performed at the ωB97X-D/def2-TZVPP level with single-point corrections computed at the DLPNO-CCSD(T)/def2-TZVPP level.
G_{sp} denotes free energy values computed at the DLPNO-CCSD(T)/def2-TZVPP//ωB97X-D/def2-TZVPP with quasi-harmonic corrections.

1

N_{imag} = 0
$G_{DLPNO-CCSD(T)}$ = −582.098861

```
C    -3.261772000000     -0.670833000000     -0.896070000000
C    -2.252121000000      0.709362000000      0.951678000000
H    -1.882138000000      1.311177000000      1.773611000000
C    -3.615793000000      0.606646000000      0.829625000000
H    -4.274948000000      1.113061000000      1.526730000000
C    -4.279350000000     -0.158769000000     -0.192555000000
H    -5.338330000000     -0.272569000000     -0.319390000000
N    -2.083256000000     -0.747774000000     -1.014904000000
B    -1.285253000000      0.009125000000     -0.000081000000
C     0.277874000000      0.003698000000     -0.008802000000
C     0.990053000000     -1.196677000000      0.139502000000
C     0.994963000000      1.201372000000     -0.153459000000
C     2.378484000000     -1.181280000000      0.148166000000
C     2.383860000000      1.179876000000     -0.151844000000
C     3.094800000000     -0.001582000000      0.003348000000
H     2.916245000000     -2.115441000000      0.268027000000
H     2.926316000000      2.110537000000     -0.277406000000
C     0.284343000000      2.520448000000     -0.322724000000
H     0.985129000000      3.308749000000     -0.594787000000
H    -0.482084000000      2.468235000000     -1.096980000000
H    -0.215190000000      2.821983000000      0.599358000000
C     0.268975000000     -2.511342000000      0.289666000000
H    -0.511488000000     -2.455106000000      1.050420000000
H    -0.215112000000     -2.802230000000     -0.644063000000
H     0.958312000000     -3.305703000000      0.572861000000
C     4.597533000000     -0.000324000000      0.041793000000
H     5.004821000000     -0.937842000000     -0.336348000000
H     5.008849000000      0.815271000000     -0.552676000000
H     4.957582000000      0.125105000000      1.065425000000
```

1-C_{Mes}

N_{imag} = 0
$G_{DLPNO-CCSD(T)}$ = −578.626414

```
C    -3.318652000000     -0.498648000000     -1.104782000000
C    -2.045062000000      0.434666000000      0.994674000000
H    -1.539632000000      0.816793000000      1.873837000000
C    -3.443327000000      0.417743000000      0.974428000000
H    -3.979648000000      0.792138000000      1.837801000000
C    -4.176739000000     -0.067869000000     -0.116619000000
H    -5.256309000000     -0.083560000000     -0.134207000000
C    -2.082682000000     -0.479116000000     -1.074914000000
```

C	-1.248451000000	-0.027770000000	-0.070082000000
C	0.233648000000	-0.012965000000	-0.044974000000
C	0.935937000000	-1.217643000000	0.067738000000
C	0.923315000000	1.200006000000	-0.138418000000
C	2.323470000000	-1.186347000000	0.097258000000
C	2.312084000000	1.186173000000	-0.109790000000
C	3.030226000000	0.005337000000	0.012898000000
H	2.867220000000	-2.119961000000	0.186568000000
H	2.847286000000	2.125276000000	-0.193169000000
C	0.190732000000	2.506454000000	-0.288166000000
H	0.881786000000	3.308359000000	-0.542840000000
H	-0.570344000000	2.448160000000	-1.066814000000
H	-0.320653000000	2.786191000000	0.634166000000
C	0.208938000000	-2.531351000000	0.153994000000
H	-0.542026000000	-2.518809000000	0.944978000000
H	-0.316952000000	-2.752468000000	-0.776567000000
H	0.902905000000	-3.346502000000	0.352062000000
C	4.532081000000	0.017892000000	0.077090000000
H	4.954239000000	-0.898555000000	-0.334494000000
H	4.945527000000	0.861864000000	-0.474487000000
H	4.873495000000	0.101696000000	1.111157000000

1_{CH3} (ASE)

N_{imag} **=** 0
$G_{DLPNO-CCSD(T)}$ **=** −621.314717

C	3.105957000000	0.000943000000	-1.437291000000
C	2.133277000000	-0.000578000000	0.906053000000
C	3.491709000000	-0.000436000000	0.706328000000
C	4.133441000000	0.000409000000	-0.586308000000
N	1.922777000000	0.001014000000	-1.568031000000
B	1.168371000000	0.000178000000	-0.289035000000
C	-0.395274000000	0.000128000000	-0.227900000000
C	-1.103127000000	1.207128000000	-0.174921000000
C	-1.103083000000	-1.206931000000	-0.175808000000
C	-2.488305000000	1.191471000000	-0.075732000000
C	-2.488268000000	-1.191394000000	-0.076609000000
C	-3.199136000000	0.000005000000	-0.023216000000
H	-3.027322000000	2.131945000000	-0.038678000000
H	-3.027252000000	-2.131914000000	-0.040253000000
C	-0.366722000000	-2.519690000000	-0.238253000000
H	-1.040823000000	-3.361855000000	-0.087378000000
H	0.121639000000	-2.648743000000	-1.206279000000
H	0.415089000000	-2.572164000000	0.522790000000
C	-0.366799000000	2.519952000000	-0.236374000000
H	0.414996000000	2.571868000000	0.524726000000
H	0.121578000000	2.649740000000	-1.204293000000
H	-1.040922000000	3.361987000000	-0.084878000000
C	-4.695920000000	-0.000065000000	0.119869000000
H	-5.138485000000	0.882959000000	-0.340816000000
H	-5.138418000000	-0.883046000000	-0.340960000000
H	-4.983990000000	-0.000157000000	1.173539000000
H	4.171099000000	-0.000970000000	1.553224000000
H	5.189977000000	0.000550000000	-0.771286000000
C	1.577486000000	-0.001462000000	2.309453000000
H	0.944284000000	-0.874851000000	2.478739000000
H	2.361442000000	-0.002014000000	3.067573000000
H	0.944407000000	0.871794000000	2.479882000000

1_{CH2} (ASE)

N_{imag} = 0

$G_{DLPNO-CCSD(T)}$ = -621.280498

C	3.048175000000	0.504911000000	-1.278258000000
C	2.106374000000	-0.357977000000	0.906616000000
C	3.622083000000	-0.420743000000	0.685159000000
C	4.104693000000	0.428341000000	-0.525191000000
N	1.865657000000	0.336190000000	-1.515963000000
B	1.134152000000	0.012069000000	-0.301843000000
C	-0.426434000000	0.017019000000	-0.207291000000
C	-1.105819000000	1.218734000000	0.027898000000
C	-1.158046000000	-1.169417000000	-0.333952000000
C	-2.489665000000	1.217101000000	0.137109000000
C	-2.542682000000	-1.137676000000	-0.225907000000
C	-3.226428000000	0.046267000000	0.014150000000
H	-3.007219000000	2.152601000000	0.319554000000
H	-3.102322000000	-2.060347000000	-0.333511000000
C	-0.451386000000	-2.471792000000	-0.605879000000
H	-1.152600000000	-3.304106000000	-0.646755000000
H	0.082358000000	-2.435702000000	-1.557756000000
H	0.285153000000	-2.688764000000	0.170528000000
C	-0.336584000000	2.507423000000	0.157960000000
H	0.420362000000	2.437818000000	0.942705000000
H	0.182777000000	2.746494000000	-0.772159000000
H	-0.993698000000	3.341253000000	0.400952000000
C	-4.722317000000	0.058488000000	0.164957000000
H	-5.149930000000	0.991048000000	-0.203147000000
H	-5.182821000000	-0.765862000000	-0.378970000000
H	-5.006257000000	-0.040401000000	1.215056000000
H	4.144593000000	-0.049343000000	1.566999000000
H	3.917406000000	-1.465311000000	0.546880000000
C	1.587945000000	-0.580121000000	2.114837000000
H	0.514784000000	-0.569154000000	2.264983000000
H	2.199940000000	-0.782514000000	2.988357000000
H	5.057663000000	0.915266000000	-0.596004000000

1-C$_{Mes,CH3}$ (ASE)

N_{imag} = 0

$G_{DLPNO-CCSD(T)}$ = -617.844157

C	3.167285000000	-0.000007000000	-1.534999000000
C	1.977805000000	0.000004000000	0.845099000000
C	3.377775000000	0.000003000000	0.734706000000
C	4.061332000000	-0.000003000000	-0.487265000000
H	5.139737000000	-0.000003000000	-0.549030000000
C	1.934881000000	-0.000006000000	-1.436273000000
C	1.149969000000	-0.000001000000	-0.304086000000
C	-0.332134000000	0.000000000000	-0.227061000000
C	-1.025072000000	1.212448000000	-0.185441000000
C	-1.025076000000	-1.212451000000	-0.185438000000
C	-2.410902000000	1.191546000000	-0.098534000000
C	-2.410901000000	-1.191548000000	-0.098533000000
C	-3.121520000000	0.000001000000	-0.050083000000
H	-2.949874000000	2.131858000000	-0.071240000000
H	-2.949876000000	-2.131859000000	-0.071241000000
C	-0.285173000000	-2.520292000000	-0.245317000000
H	-0.972606000000	-3.361523000000	-0.174424000000
H	0.273659000000	-2.612487000000	-1.177991000000
H	0.441764000000	-2.604765000000	0.564520000000
C	-0.285171000000	2.520290000000	-0.245330000000
H	0.441785000000	2.604760000000	0.564490000000

H	0.273640000000	2.612487000000	-1.178018000000
H	-0.972602000000	3.361521000000	-0.174421000000
C	-4.619501000000	0.000002000000	0.078144000000
H	-5.057262000000	0.883005000000	-0.386752000000
H	-5.057303000000	-0.882782000000	-0.387132000000
H	-4.917341000000	-0.000220000000	1.128950000000
C	1.328471000000	0.000009000000	2.205782000000
H	0.689738000000	-0.874903000000	2.336548000000
H	2.072423000000	0.000020000000	2.999801000000
H	0.689724000000	0.874912000000	2.336536000000
H	3.961982000000	0.000008000000	1.647178000000

1-$C_{Mes,CH2}$ (ASE)

N_{imag} = 0
$G_{DLPNO-CCSD(T)}$ = −617.788959

C	3.147340000000	0.355326000000	-1.458082000000
C	1.960927000000	-0.176915000000	0.911363000000
C	3.486142000000	-0.281327000000	0.765917000000
C	4.100036000000	0.164486000000	-0.573862000000
H	5.171810000000	0.235083000000	-0.689577000000
C	1.882676000000	0.282176000000	-1.351515000000
C	1.098184000000	0.035622000000	-0.314553000000
C	-0.379694000000	0.025249000000	-0.223275000000
C	-1.075038000000	1.227147000000	-0.066252000000
C	-1.069121000000	-1.189459000000	-0.277299000000
C	-2.460418000000	1.193657000000	0.026694000000
C	-2.454293000000	-1.180164000000	-0.182839000000
C	-3.167820000000	0.000716000000	-0.028396000000
H	-3.001058000000	2.125851000000	0.145546000000
H	-2.990302000000	-2.121239000000	-0.232680000000
C	-0.325549000000	-2.485369000000	-0.444073000000
H	-1.010443000000	-3.331695000000	-0.444367000000
H	0.234820000000	-2.498132000000	-1.380098000000
H	0.395168000000	-2.628869000000	0.362942000000
C	-0.339592000000	2.536967000000	-0.001229000000
H	0.443778000000	2.510188000000	0.757873000000
H	0.145054000000	2.762442000000	-0.952472000000
H	-1.019117000000	3.353915000000	0.236185000000
C	-4.665131000000	-0.015078000000	0.106750000000
H	-5.107338000000	0.904684000000	-0.275326000000
H	-5.103129000000	-0.854115000000	-0.433345000000
H	-4.957600000000	-0.109995000000	1.154775000000
C	1.390407000000	-0.270915000000	2.113641000000
H	0.315309000000	-0.241636000000	2.225914000000
H	1.981493000000	-0.385997000000	3.012627000000
H	3.956414000000	0.275485000000	1.578151000000
H	3.770801000000	-1.328668000000	0.915911000000

Furan

N_{imag} = 0
$G_{DLPNO-CCSD(T)}$ = −229.592672

O	0.000029000000	-1.150096000000	0.000301000000
C	0.714975000000	0.952542000000	0.000311000000
C	-0.715003000000	0.952517000000	-0.000177000000
C	-1.085554000000	-0.346485000000	-0.000058000000
H	-2.040336000000	-0.841372000000	-0.000090000000
C	1.085546000000	-0.346461000000	-0.000409000000
H	2.040376000000	-0.841262000000	-0.000577000000
H	1.371294000000	1.805385000000	0.000563000000

H	-1.371355000000	1.805337000000	-0.000302000000

1-C$_H$

***N*$_{imag}$ = 0**
***G*$_{DLPNO-CCSD(T)}$ = -230.428192**

C	-0.618506000000	-1.227341000000	0.000062000000
C	0.700122000000	1.049089000000	0.000027000000
H	1.222296000000	1.997774000000	0.000096000000
C	-0.699858000000	1.049239000000	0.000013000000
H	-1.221803000000	1.998051000000	0.000089000000
C	-1.453796000000	-0.132249000000	-0.000089000000
H	-2.533708000000	-0.134106000000	-0.000054000000
C	0.618149000000	-1.227328000000	0.000019000000
C	1.453805000000	-0.132585000000	-0.000053000000
H	2.533712000000	-0.134667000000	0.000003000000

TS-C$_H$

***N*$_{imag}$ = 1, -195 cm^{-1}**
***G*$_{DLPNO-CCSD(T)}$ = -460.000819**

O	1.882319000000	-0.000003000000	-1.158100000000
C	2.139941000000	-0.697149000000	0.944722000000
C	2.139939000000	0.697155000000	0.944718000000
C	1.869981000000	1.076356000000	-0.352246000000
H	1.873154000000	2.036526000000	-0.836526000000
C	1.869985000000	-1.076357000000	-0.352240000000
H	1.873162000000	-2.036530000000	-0.836515000000
C	-0.541128000000	-0.631498000000	-0.094310000000
C	-2.843838000000	0.698053000000	0.094826000000
H	-3.786744000000	1.224763000000	0.176340000000
C	-2.843840000000	-0.698051000000	0.094827000000
H	-3.786746000000	-1.224759000000	0.176340000000
C	-1.656934000000	-1.433947000000	-0.006764000000
H	-1.661496000000	-2.516001000000	-0.012540000000
H	2.237661000000	-1.358721000000	1.787749000000
H	2.237656000000	1.358732000000	1.787742000000
C	-0.541127000000	0.631494000000	-0.094310000000
C	-1.656931000000	1.433947000000	-0.006764000000
H	-1.661491000000	2.516001000000	-0.012540000000

RC

***N*$_{imag}$ = 0**
***G*$_{DLPNO-CCSD(T)}$ = -808.208160**

O	3.176962000000	-1.291190000000	1.656708000000
C	4.164448000000	-1.095370000000	-0.324487000000
C	3.237603000000	-2.172696000000	-0.383242000000
C	2.659075000000	-2.228782000000	0.838877000000
H	1.895867000000	-2.856484000000	1.262785000000
C	4.074850000000	-0.592003000000	0.936275000000
H	4.604212000000	0.185299000000	1.458098000000
C	2.226193000000	0.971496000000	-0.145163000000
C	0.289948000000	2.504465000000	-0.780621000000
H	-0.486626000000	3.199900000000	-1.078077000000
C	1.586189000000	2.952695000000	-0.873524000000
H	1.799154000000	3.957772000000	-1.222790000000
C	2.728565000000	2.155411000000	-0.539134000000
H	3.755227000000	2.460746000000	-0.609183000000
H	4.810058000000	-0.738401000000	-1.108172000000

H	3.011100000000	-2.799149000000	-1.227816000000
N	1.205626000000	0.374023000000	0.015399000000
B	-0.047107000000	1.094293000000	-0.305302000000
C	-1.445269000000	0.400513000000	-0.156376000000
C	-1.723780000000	-0.796080000000	-0.837742000000
C	-2.443646000000	0.957560000000	0.656673000000
C	-2.967780000000	-1.397894000000	-0.704439000000
C	-3.674598000000	0.323130000000	0.777871000000
C	-3.958749000000	-0.853141000000	0.100711000000
H	-3.169835000000	-2.318674000000	-1.241176000000
H	-4.431596000000	0.758700000000	1.421045000000
C	-2.208796000000	2.237937000000	1.417986000000
H	-2.992387000000	2.400462000000	2.157287000000
H	-1.249722000000	2.229689000000	1.936456000000
H	-2.200096000000	3.099236000000	0.747929000000
C	-0.689897000000	-1.443316000000	-1.723951000000
H	-0.279417000000	-0.731757000000	-2.442522000000
H	0.150654000000	-1.819463000000	-1.138723000000
H	-1.119229000000	-2.275676000000	-2.280581000000
C	-5.308590000000	-1.505785000000	0.211433000000
H	-5.231309000000	-2.591032000000	0.143051000000
H	-5.793660000000	-1.257109000000	1.155139000000
H	-5.966541000000	-1.173013000000	-0.594566000000

RC–C_{Mes}

N_{imag} **=** 0
$G_{DLPNO\text{-}CCSD(T)}$ **=** −811.678907

O	1.814061000000	2.509880000000	-1.240795000000
C	2.041653000000	2.931743000000	0.928250000000
C	0.713693000000	2.467122000000	0.688238000000
C	0.639397000000	2.224047000000	-0.640654000000
H	-0.152941000000	1.869088000000	-1.275416000000
C	2.658330000000	2.929076000000	-0.274589000000
H	3.652566000000	3.185983000000	-0.593126000000
C	2.972208000000	-0.842999000000	-0.019918000000
C	1.024354000000	-2.640463000000	0.153991000000
H	0.243450000000	-3.388479000000	0.226538000000
C	2.362319000000	-3.049938000000	0.162954000000
H	2.579424000000	-4.108133000000	0.243689000000
C	3.427640000000	-2.145127000000	0.072827000000
H	4.459124000000	-2.467529000000	0.080185000000
H	2.478493000000	3.217117000000	1.869336000000
H	-0.079530000000	2.325580000000	1.401660000000
C	1.775832000000	-0.524109000000	-0.026027000000
C	0.637250000000	-1.291402000000	0.054448000000
C	-0.769261000000	-0.824498000000	0.039925000000
C	-1.390963000000	-0.461391000000	1.237342000000
C	-1.447455000000	-0.704466000000	-1.175341000000
C	-2.693950000000	0.017022000000	1.198540000000
C	-2.750340000000	-0.222004000000	-1.172138000000
C	-3.391281000000	0.141885000000	0.004180000000
H	-3.176128000000	0.304329000000	2.126161000000
H	-3.276031000000	-0.122636000000	-2.115060000000
C	-0.770212000000	-1.060476000000	-2.470048000000
H	-0.456755000000	-2.105165000000	-2.480122000000
H	-1.434225000000	-0.894642000000	-3.316717000000
H	0.131642000000	-0.462890000000	-2.618342000000
C	-0.654217000000	-0.564686000000	2.543696000000
H	-0.348315000000	-1.592210000000	2.745474000000
H	0.257415000000	0.035705000000	2.525853000000

H	-1.275198000000	-0.224558000000	3.370892000000
C	-4.811641000000	0.634027000000	-0.010195000000
H	-5.508621000000	-0.194134000000	0.135088000000
H	-4.989146000000	1.355300000000	0.787258000000
H	-5.057849000000	1.108066000000	-0.959881000000

TS

N_{imag} = 1, -197 cm^{-1}
$G_{DLPNO-CCSD(T)}$ = −811.676080

O	3.038057000000	-1.098336000000	1.697978000000
C	3.869779000000	-1.255872000000	-0.368479000000
C	2.936219000000	-2.284571000000	-0.186840000000
C	2.427168000000	-2.101081000000	1.067669000000
H	1.672418000000	-2.626718000000	1.626030000000
C	3.834923000000	-0.491556000000	0.784864000000
H	4.509034000000	0.254422000000	1.167138000000
C	2.362218000000	0.744573000000	-0.094303000000
C	0.470274000000	2.369722000000	-0.897471000000
H	-0.270659000000	3.073943000000	-1.260756000000
C	1.788459000000	2.746432000000	-0.995586000000
H	2.057430000000	3.714591000000	-1.406245000000
C	2.861970000000	1.908176000000	-0.584061000000
H	3.902940000000	2.174803000000	-0.644433000000
H	4.450584000000	-1.035770000000	-1.247631000000
H	2.622778000000	-3.026070000000	-0.899712000000
N	1.261294000000	0.242983000000	0.052564000000
B	0.082556000000	1.009170000000	-0.330989000000
C	-1.364030000000	0.405939000000	-0.171756000000
C	-1.742465000000	-0.734700000000	-0.895444000000
C	-2.299864000000	0.989042000000	0.694251000000
C	-3.020505000000	-1.260278000000	-0.751766000000
C	-3.568308000000	0.435463000000	0.824193000000
C	-3.950307000000	-0.687761000000	0.104937000000
H	-3.298644000000	-2.140177000000	-1.322408000000
H	-4.277130000000	0.892251000000	1.506700000000
C	-1.943701000000	2.209225000000	1.505322000000
H	-2.741692000000	2.464634000000	2.201957000000
H	-1.029447000000	2.051798000000	2.079762000000
H	-1.766907000000	3.073232000000	0.863130000000
C	-0.774039000000	-1.400144000000	-1.840539000000
H	-0.375022000000	-0.686645000000	-2.564089000000
H	0.080814000000	-1.809760000000	-1.299950000000
H	-1.253767000000	-2.208995000000	-2.391102000000
C	-5.339725000000	-1.250065000000	0.227002000000
H	-5.350294000000	-2.325167000000	0.046784000000
H	-5.756425000000	-1.066703000000	1.217471000000
H	-6.010701000000	-0.788970000000	-0.501448000000

TS-C_{Mes}

N_{imag} = 1, -190 cm^{-1}
$G_{DLPNO-CCSD(T)}$ = −808.201029

O	-2.364111000000	-1.883363000000	-1.187700000000
C	-2.965797000000	-2.216151000000	0.931964000000
C	-1.570219000000	-2.132522000000	0.882473000000
C	-1.251312000000	-1.818207000000	-0.421766000000
H	-0.307619000000	-1.806499000000	-0.939674000000
C	-3.399941000000	-1.984793000000	-0.346792000000
H	-4.379516000000	-1.979882000000	-0.790005000000

C	-2.734225000000	0.729754000000	-0.068657000000
C	-0.694049000000	2.509616000000	0.137328000000
H	0.111069000000	3.229679000000	0.224055000000
C	-2.018608000000	2.951736000000	0.142382000000
H	-2.212609000000	4.013279000000	0.240112000000
C	-3.094934000000	2.065807000000	0.031870000000
H	-4.114599000000	2.430290000000	0.038076000000
H	-3.589769000000	-2.363217000000	1.795771000000
H	-0.869322000000	-2.205564000000	1.696179000000
C	-1.514226000000	0.397142000000	-0.081556000000
C	-0.369161000000	1.149883000000	0.021432000000
C	1.015965000000	0.617636000000	0.008986000000
C	1.651915000000	0.305324000000	1.215065000000
C	1.659842000000	0.382240000000	-1.208244000000
C	2.929375000000	-0.236698000000	1.181747000000
C	2.939773000000	-0.160810000000	-1.199932000000
C	3.591604000000	-0.474604000000	-0.016231000000
H	3.420786000000	-0.483475000000	2.116416000000
H	3.438212000000	-0.345072000000	-2.144882000000
C	0.977806000000	0.702732000000	-2.510185000000
H	0.716130000000	1.760169000000	-2.566602000000
H	1.618471000000	0.461541000000	-3.356834000000
H	0.043371000000	0.148536000000	-2.617705000000
C	0.963212000000	0.550742000000	2.529829000000
H	0.813035000000	1.617901000000	2.700638000000
H	-0.026868000000	0.092988000000	2.550969000000
H	1.548167000000	0.152903000000	3.357677000000
C	4.988708000000	-1.030156000000	-0.020949000000
H	5.712926000000	-0.255495000000	0.240457000000
H	5.097196000000	-1.834994000000	0.706437000000
H	5.259459000000	-1.418192000000	-1.002069000000

2

N_{imag} = 0

$G_{DLPNO-CCSD(T)}$ = −811.739699

O	-2.437882000000	-1.511120000000	-1.415500000000
C	-3.145573000000	-1.918463000000	0.723545000000
C	-1.846116000000	-2.170219000000	0.686685000000
C	-1.342900000000	-1.400149000000	-0.535354000000
H	-0.398636000000	-1.674982000000	-0.989522000000
C	-3.406582000000	-1.015668000000	-0.485614000000
H	-4.403651000000	-0.981954000000	-0.907434000000
C	-2.747642000000	0.316174000000	-0.139348000000
C	-0.937864000000	2.355763000000	0.480548000000
H	-0.291467000000	3.190691000000	0.725897000000
C	-2.285047000000	2.568546000000	0.488569000000
H	-2.684904000000	3.547235000000	0.733461000000
C	-3.222568000000	1.538335000000	0.180026000000
H	-4.285818000000	1.728447000000	0.192190000000
H	-3.873332000000	-2.182091000000	1.473635000000
H	-1.226642000000	-2.701824000000	1.390320000000
N	-1.413433000000	0.039474000000	-0.175734000000
B	-0.388680000000	0.979007000000	0.128694000000
C	1.116314000000	0.515730000000	0.070594000000
C	1.776862000000	0.099052000000	1.232657000000
C	1.805479000000	0.485720000000	-1.148538000000
C	3.096717000000	-0.330060000000	1.163545000000
C	3.125223000000	0.053163000000	-1.187365000000
C	3.789622000000	-0.356866000000	-0.039149000000
H	3.597265000000	-0.652534000000	2.070207000000

H	3.648794000000	0.034530000000	-2.137166000000
C	1.115766000000	0.915456000000	-2.417865000000
H	1.798849000000	0.902047000000	-3.266135000000
H	0.273576000000	0.261456000000	-2.655943000000
H	0.711688000000	1.925115000000	-2.322832000000
C	1.057375000000	0.109906000000	2.557176000000
H	0.165066000000	-0.519916000000	2.530406000000
H	1.698159000000	-0.249216000000	3.361561000000
H	0.721750000000	1.116426000000	2.813780000000
C	5.228804000000	-0.789317000000	-0.093468000000
H	5.463104000000	-1.491072000000	0.706838000000
H	5.464093000000	-1.266360000000	-1.044894000000
H	5.895378000000	0.068997000000	0.016440000000

2-C_{Mes}

N_{imag} = 0
$G_{DLPNO-CCSD(T)}$ = −808.298245

O	-2.553144000000	-1.209700000000	-1.619185000000
C	-3.255847000000	-1.914209000000	0.434937000000
C	-1.983217000000	-2.261726000000	0.324649000000
C	-1.411047000000	-1.349678000000	-0.768251000000
H	-0.537878000000	-1.691892000000	-1.312920000000
C	-3.458533000000	-0.792158000000	-0.593879000000
H	-4.458648000000	-0.633801000000	-0.982565000000
C	-2.701674000000	0.424526000000	-0.056443000000
C	-0.707809000000	2.124372000000	0.762741000000
H	0.070820000000	2.805263000000	1.083271000000
C	-2.033553000000	2.497812000000	0.872298000000
H	-2.279623000000	3.470665000000	1.277175000000
C	-3.062436000000	1.639029000000	0.464420000000
H	-4.098478000000	1.938407000000	0.555262000000
H	-3.997456000000	-2.260681000000	1.136617000000
H	-1.415795000000	-2.966692000000	0.910767000000
C	-1.363178000000	0.054848000000	-0.167731000000
C	-0.339988000000	0.873766000000	0.238990000000
C	1.082609000000	0.450370000000	0.129963000000
C	1.705741000000	-0.174809000000	1.213464000000
C	1.780428000000	0.649749000000	-1.064628000000
C	3.027914000000	-0.582138000000	1.087973000000
C	3.101393000000	0.228842000000	-1.151098000000
C	3.743927000000	-0.385546000000	-0.084839000000
H	3.509912000000	-1.069837000000	1.927909000000
H	3.641919000000	0.382244000000	-2.078408000000
C	1.110332000000	1.301052000000	-2.243681000000
H	0.733162000000	2.291713000000	-1.985788000000
H	1.802424000000	1.404319000000	-3.077824000000
H	0.251261000000	0.719366000000	-2.582916000000
C	0.955738000000	-0.411623000000	2.496473000000
H	0.649540000000	0.530031000000	2.954846000000
H	0.042148000000	-0.983091000000	2.322631000000
H	1.569423000000	-0.955546000000	3.212827000000
C	5.184575000000	-0.803153000000	-0.189300000000
H	5.846107000000	0.011605000000	0.113109000000
H	5.398426000000	-1.655621000000	0.455039000000
H	5.445639000000	-1.074794000000	-1.211900000000

1-B_{DD}

N_{imag} = 0
$G_{DLPNO-CCSD(T)}$ = −367.610045

C	-1.753300000000	-1.131381000000	-0.000049000000
C	-0.809584000000	1.204751000000	-0.000042000000
H	-0.484285000000	2.238087000000	0.000151000000
C	-2.157866000000	1.003026000000	0.000090000000
H	-2.841729000000	1.845187000000	0.000290000000
C	-2.798458000000	-0.302075000000	0.000111000000
H	-3.852015000000	-0.498152000000	0.000834000000
N	-0.572815000000	-1.274567000000	-0.000268000000
B	0.188707000000	0.021096000000	-0.000241000000
N	1.593009000000	0.019227000000	-0.000513000000
C	2.376252000000	1.232128000000	0.000086000000
H	3.022568000000	1.281404000000	-0.882775000000
H	1.733578000000	2.108380000000	-0.002417000000
H	3.018656000000	1.283468000000	0.885722000000
C	2.391688000000	-1.183799000000	0.000344000000
H	3.038028000000	-1.224513000000	-0.882956000000
H	3.035465000000	-1.225196000000	0.885509000000
H	1.752451000000	-2.062660000000	-0.000920000000

1-B$_D$

N_{imag} = 0
$G_{DLPNO-CCSD(T)}$ = -807.428741

C	-3.638795000000	-1.166417000000	-0.672965000000
C	-2.808686000000	0.780105000000	0.701518000000
H	-2.517775000000	1.630562000000	1.304869000000
C	-4.151535000000	0.517878000000	0.608645000000
H	-4.872636000000	1.139616000000	1.128990000000
C	-4.717987000000	-0.570472000000	-0.148037000000
H	-5.757990000000	-0.820403000000	-0.227071000000
N	-2.456599000000	-1.144373000000	-0.756931000000
B	-1.750053000000	-0.075065000000	0.000472000000
C	-0.198059000000	0.058058000000	-0.001135000000
C	0.645778000000	-1.055403000000	0.058518000000
C	0.431392000000	1.313833000000	-0.055854000000
C	2.037538000000	-0.948199000000	0.055419000000
C	1.810501000000	1.454922000000	-0.062458000000
C	2.604500000000	0.316109000000	-0.007178000000
H	2.656628000000	-1.826579000000	0.107941000000
H	2.299019000000	2.414415000000	-0.113448000000
O	-0.397574000000	2.380610000000	-0.121161000000
C	0.152833000000	3.671924000000	-0.195451000000
H	0.759417000000	3.902206000000	0.684751000000
H	0.761960000000	3.797403000000	-1.094765000000
H	-0.690811000000	4.356067000000	-0.237508000000
O	3.938338000000	0.538964000000	-0.017182000000
C	4.807244000000	-0.566375000000	0.032707000000
H	4.672767000000	-1.140869000000	0.953479000000
H	4.666792000000	-1.226021000000	-0.828094000000
H	5.815216000000	-0.160547000000	0.010092000000
O	0.029038000000	-2.255822000000	0.143604000000
C	0.799718000000	-3.429105000000	0.183160000000
H	1.414200000000	-3.538797000000	-0.714975000000
H	1.443155000000	-3.460067000000	1.067282000000
H	0.091446000000	-4.252218000000	0.231201000000

1-B$_A$

N_{imag} = 0
$G_{DLPNO-CCSD(T)}$ = -761.873614

C	-3.635435000000	-1.127643000000	0.000612000000

C	-2.674820000000	1.196323000000	-0.001157000000
H	-2.333984000000	2.224991000000	-0.001977000000
C	-4.035030000000	1.009238000000	-0.001473000000
H	-4.712961000000	1.855946000000	-0.002385000000
C	-4.671584000000	-0.282634000000	-0.000413000000
H	-5.726729000000	-0.475066000000	-0.000508000000
N	-2.454960000000	-1.261966000000	0.001365000000
B	-1.701860000000	0.023458000000	0.000685000000
C	-0.143929000000	0.014233000000	0.001144000000
C	0.563773000000	-1.188454000000	0.000535000000
C	0.574463000000	1.211218000000	0.001017000000
C	1.941271000000	-1.186649000000	-0.000194000000
C	1.952176000000	1.196930000000	0.000517000000
C	2.654566000000	0.002027000000	-0.000236000000
H	0.047244000000	-2.138645000000	0.001108000000
H	0.075025000000	2.170361000000	0.001660000000
F	2.621578000000	-2.332280000000	-0.000952000000
F	2.641885000000	2.337162000000	0.000533000000
F	3.980172000000	-0.003620000000	-0.001023000000

1-B$_{AA}$

***N*$_{imag}$ =** 0
***G*$_{DLPNO-CCSD(T)}$ =** −960.144842

C	-3.661512000000	-1.032359000000	-0.465199000000
C	-2.675878000000	1.083583000000	0.485393000000
H	-2.322515000000	2.015302000000	0.906907000000
C	-4.038985000000	0.919022000000	0.414816000000
H	-4.707632000000	1.695420000000	0.770525000000
C	-4.688512000000	-0.251714000000	-0.109985000000
H	-5.745884000000	-0.421072000000	-0.173271000000
N	-2.481203000000	-1.142329000000	-0.520523000000
B	-1.715656000000	0.007522000000	0.001446000000
C	-0.146378000000	-0.000191000000	0.000785000000
C	0.596418000000	-1.174942000000	0.076700000000
C	0.591175000000	1.176862000000	-0.072525000000
C	1.980314000000	-1.189387000000	0.080106000000
C	1.974666000000	1.200333000000	-0.077300000000
C	2.672354000000	0.007197000000	0.000843000000
F	-0.015975000000	-2.352008000000	0.168429000000
F	-0.028844000000	2.352951000000	-0.159266000000
F	2.648140000000	-2.332295000000	0.162167000000
F	2.635677000000	2.347452000000	-0.157950000000
F	3.994418000000	0.010413000000	0.001117000000

TS-B$_{DD}$

***N*$_{imag}$ =** 1,-224 cm^{-1}
***G*$_{DLPNO-CCSD(T)}$ =** −597.182482

O	-2.358324000000	-0.798256000000	-1.131379000000
C	-2.783115000000	0.103402000000	0.871478000000
C	-2.470404000000	-1.245853000000	1.051945000000
C	-2.165455000000	-1.720636000000	-0.195229000000
H	-1.852563000000	-2.691462000000	-0.538895000000
C	-2.588982000000	0.373444000000	-0.479625000000
H	-2.946614000000	1.183612000000	-1.090285000000
C	-0.698496000000	0.948406000000	-0.120215000000
C	1.808400000000	1.761432000000	0.075606000000
H	2.810427000000	2.163580000000	0.177934000000
C	0.792187000000	2.672955000000	0.047120000000
H	1.007076000000	3.735586000000	0.105626000000

C	-0.585952000000	2.301037000000	-0.045318000000
H	-1.403462000000	3.000506000000	-0.078078000000
H	-3.027336000000	0.834213000000	1.622993000000
H	-2.403865000000	-1.792043000000	1.975656000000
N	0.064218000000	-0.012836000000	-0.093468000000
B	1.501244000000	0.251123000000	-0.005536000000
N	2.430294000000	-0.823791000000	0.005163000000
C	2.032918000000	-2.207071000000	-0.038311000000
H	2.379901000000	-2.749893000000	0.849980000000
H	0.949237000000	-2.281003000000	-0.083345000000
H	2.452010000000	-2.714910000000	-0.915902000000
C	3.856953000000	-0.640362000000	0.068639000000
H	4.354637000000	-1.101093000000	-0.793466000000
H	4.111499000000	0.416147000000	0.075721000000
H	4.279515000000	-1.096935000000	0.972374000000

TS-B$_D$

***N*$_{imag}$ =** 1, -214 cm^{-1}
***G*$_{DLPNO\text{-}CCSD(T)}$ =** -1037.002751

O	3.451477000000	-0.787503000000	1.913205000000
C	4.278660000000	-1.383054000000	-0.075048000000
C	3.272429000000	-2.288714000000	0.274320000000
C	2.765958000000	-1.835503000000	1.461382000000
H	1.967736000000	-2.191294000000	2.088683000000
C	4.285249000000	-0.409301000000	0.911757000000
H	5.013367000000	0.341909000000	1.162413000000
C	2.893475000000	0.716064000000	-0.169175000000
C	1.065141000000	2.237473000000	-1.277462000000
H	0.349972000000	2.894778000000	-1.758741000000
C	2.395567000000	2.545013000000	-1.421687000000
H	2.701792000000	3.416481000000	-1.992207000000
C	3.436466000000	1.755291000000	-0.854614000000
H	4.487092000000	1.969759000000	-0.950024000000
H	4.875145000000	-1.367986000000	-0.970948000000
H	2.902696000000	-3.119564000000	-0.298712000000
N	1.770307000000	0.294670000000	0.048575000000
B	0.625522000000	1.010390000000	-0.485899000000
C	-0.844826000000	0.502932000000	-0.244104000000
C	-1.230935000000	-0.809081000000	-0.510856000000
C	-1.846005000000	1.355834000000	0.241326000000
C	-2.533518000000	-1.273379000000	-0.308860000000
C	-3.150020000000	0.931848000000	0.457564000000
C	-3.482283000000	-0.387005000000	0.178420000000
H	-2.797535000000	-2.292496000000	-0.532099000000
H	-3.920951000000	1.582674000000	0.837718000000
O	-0.259213000000	-1.621039000000	-1.006236000000
C	-0.579531000000	-2.940771000000	-1.350654000000
H	-0.907664000000	-3.521774000000	-0.482539000000
H	-1.354657000000	-2.984489000000	-2.121751000000
H	0.333074000000	-3.380813000000	-1.746861000000
O	-4.774448000000	-0.724534000000	0.414768000000
C	-5.182235000000	-2.042903000000	0.151216000000
H	-4.631710000000	-2.764497000000	0.762094000000
H	-6.237501000000	-2.092026000000	0.407807000000
H	-5.058826000000	-2.298963000000	-0.905203000000
O	-1.450338000000	2.624937000000	0.503246000000
C	-2.393782000000	3.548390000000	0.981095000000
H	-2.805487000000	3.241294000000	1.946962000000
H	-1.860736000000	4.487957000000	1.103557000000
H	-3.214260000000	3.690731000000	0.271743000000

TS-B_A

N_{imag} = 1, -150 cm^{-1}
$G_{DLPNO-CCSD(T)}$ = -991.452198

O	-3.352251000000	-1.519912000000	-1.141092000000
C	-4.160246000000	-0.885483000000	0.839543000000
C	-3.273937000000	-1.956838000000	1.043417000000
C	-2.785683000000	-2.267459000000	-0.190216000000
H	-2.072876000000	-3.000940000000	-0.524744000000
C	-4.122274000000	-0.603287000000	-0.511633000000
H	-4.758661000000	-0.006525000000	-1.140424000000
C	-2.607231000000	0.856212000000	-0.076960000000
C	-0.733041000000	2.676143000000	0.065273000000
H	-0.004304000000	3.476188000000	0.136964000000
C	-2.055635000000	3.050418000000	0.060692000000
H	-2.334513000000	4.097757000000	0.114935000000
C	-3.121120000000	2.110288000000	-0.008132000000
H	-4.165290000000	2.369470000000	-0.019391000000
H	-4.721786000000	-0.340977000000	1.578974000000
H	-2.985142000000	-2.405954000000	1.976661000000
N	-1.508607000000	0.331358000000	-0.070403000000
B	-0.347163000000	1.207056000000	-0.007422000000
C	1.094045000000	0.584592000000	-0.004810000000
C	1.261814000000	-0.800588000000	-0.002725000000
C	2.235302000000	1.387365000000	-0.001670000000
C	2.522146000000	-1.355358000000	0.003145000000
C	3.491064000000	0.819029000000	0.003025000000
C	3.654606000000	-0.556921000000	0.005794000000
H	0.402227000000	-1.456942000000	-0.004358000000
F	4.867832000000	-1.097508000000	0.010700000000
H	2.164341000000	2.466523000000	-0.004870000000
F	2.681170000000	-2.681918000000	0.006375000000
F	4.583355000000	1.585787000000	0.004755000000

TS-B_{AA}

N_{imag} = 1, -96 cm^{-1}
$G_{DLPNO-CCSD(T)}$ = -1189.725350

O	-3.544096000000	-1.716887000000	-0.605109000000
C	-4.329978000000	-0.614166000000	1.163885000000
C	-3.413233000000	-1.586832000000	1.614073000000
C	-2.942450000000	-2.194899000000	0.492780000000
H	-2.211051000000	-2.967108000000	0.333394000000
C	-4.337917000000	-0.701504000000	-0.207527000000
H	-4.969546000000	-0.265603000000	-0.960849000000
C	-2.718374000000	0.892822000000	-0.175691000000
C	-0.789776000000	2.583608000000	-0.602186000000
H	-0.031194000000	3.333807000000	-0.785730000000
C	-2.101151000000	2.997550000000	-0.666930000000
H	-2.340319000000	4.029929000000	-0.899915000000
C	-3.205025000000	2.127812000000	-0.440240000000
H	-4.239396000000	2.416968000000	-0.490697000000
H	-4.893404000000	0.087731000000	1.754250000000
H	-3.098457000000	-1.777539000000	2.624365000000
N	-1.648117000000	0.341806000000	-0.063448000000
B	-0.455119000000	1.138894000000	-0.280549000000
C	0.975299000000	0.476685000000	-0.162784000000
C	2.088030000000	1.194276000000	0.260121000000
C	1.208211000000	-0.859750000000	-0.469565000000
C	3.349218000000	0.635648000000	0.380517000000

C	2.454623000000	-1.452650000000	-0.366369000000
C	3.532675000000	-0.698566000000	0.063273000000
F	1.971960000000	2.480344000000	0.592029000000
F	0.215346000000	-1.637619000000	-0.903949000000
F	4.379057000000	1.361827000000	0.798833000000
F	2.627369000000	-2.732858000000	-0.677292000000
F	4.731560000000	-1.250629000000	0.169580000000

2-B$_{DD}$

***N*$_{imag}$ =** 0
***G*$_{DLPNO-CCSD(T)}$ =** -597.237017

O	1.985469000000	-0.827525000000	1.264414000000
C	2.614406000000	-0.794491000000	-0.935650000000
C	1.627778000000	-1.670698000000	-0.822056000000
C	0.911100000000	-1.269536000000	0.470102000000
H	0.296054000000	-1.991699000000	0.989934000000
C	2.488810000000	0.099332000000	0.297826000000
H	3.364938000000	0.629217000000	0.651273000000
C	1.224829000000	0.922793000000	0.087728000000
C	-1.378604000000	1.894020000000	-0.149237000000
H	-2.356528000000	2.349037000000	-0.256459000000
C	-0.307640000000	2.720944000000	-0.224214000000
H	-0.446890000000	3.786521000000	-0.374334000000
C	1.036848000000	2.238905000000	-0.114016000000
H	1.880947000000	2.910779000000	-0.163094000000
H	3.302530000000	-0.641710000000	-1.751097000000
H	1.302645000000	-2.428041000000	-1.517224000000
N	0.206538000000	0.009845000000	0.178902000000
B	-1.184539000000	0.373932000000	0.020134000000
N	-2.239696000000	-0.583327000000	0.002147000000
C	-2.080954000000	-1.995757000000	-0.247551000000
H	-2.865079000000	-2.344996000000	-0.926689000000
H	-1.130481000000	-2.206907000000	-0.733339000000
H	-2.149255000000	-2.600849000000	0.665357000000
C	-3.623799000000	-0.206701000000	0.176994000000
H	-4.091333000000	-0.809481000000	0.963264000000
H	-3.709481000000	0.835368000000	0.471400000000
H	-4.203657000000	-0.355184000000	-0.741871000000

2-B$_{D}$

***N*$_{imag}$ =** 0
***G*$_{DLPNO-CCSD(T)}$ =** -1037.068268

O	2.956057000000	-0.519829000000	2.013336000000
C	3.724170000000	-1.944712000000	0.396149000000
C	2.433861000000	-2.192707000000	0.556558000000
C	1.875079000000	-0.922150000000	1.200313000000
H	0.933154000000	-0.961560000000	1.733316000000
C	3.924665000000	-0.536876000000	0.959573000000
H	4.910480000000	-0.245617000000	1.301505000000
C	3.226168000000	0.396139000000	-0.024267000000
C	1.373422000000	1.807065000000	-1.562136000000
H	0.703246000000	2.394793000000	-2.178222000000
C	2.712664000000	2.013428000000	-1.697682000000
H	3.088564000000	2.743503000000	-2.407338000000
C	3.673260000000	1.302169000000	-0.917675000000
H	4.731668000000	1.491069000000	-1.023597000000
H	4.476170000000	-2.527531000000	-0.110512000000

H	1.848899000000	-3.033661000000	0.226564000000
N	1.899909000000	0.129588000000	0.153880000000
B	0.854565000000	0.781118000000	-0.560909000000
C	-0.656007000000	0.433532000000	-0.277011000000
C	-1.185402000000	-0.833988000000	-0.491635000000
C	-1.550436000000	1.415651000000	0.169312000000
C	-2.531681000000	-1.140604000000	-0.283488000000
C	-2.893917000000	1.152064000000	0.391514000000
C	-3.374353000000	-0.131387000000	0.160132000000
H	-2.907437000000	-2.132508000000	-0.465420000000
H	-3.586370000000	1.901206000000	0.740596000000
O	-0.297846000000	-1.767095000000	-0.933763000000
C	-0.757839000000	-3.053723000000	-1.257893000000
H	-1.163720000000	-3.572331000000	-0.383974000000
H	-1.517935000000	-3.023492000000	-2.043200000000
H	0.107238000000	-3.600445000000	-1.626373000000
O	-4.695290000000	-0.308293000000	0.399235000000
C	-5.252626000000	-1.581847000000	0.188360000000
H	-4.790222000000	-2.334533000000	0.833395000000
H	-6.306702000000	-1.497290000000	0.439578000000
H	-5.157489000000	-1.894160000000	-0.855556000000
O	-1.003275000000	2.635407000000	0.380178000000
C	-1.829592000000	3.688805000000	0.808599000000
H	-2.277397000000	3.476702000000	1.783473000000
H	-1.186458000000	4.560850000000	0.893185000000
H	-2.623667000000	3.895641000000	0.085740000000

2-B$_A$

***N*$_{imag}$ =** 0
***G*$_{DLPNO-CCSD(T)}$ =** -991.508758

O	-2.883350000000	-1.138905000000	-1.580838000000
C	-3.613411000000	-1.778652000000	0.492424000000
C	-2.339580000000	-2.127313000000	0.400396000000
C	-1.781009000000	-1.239009000000	-0.713167000000
H	-0.869363000000	-1.524083000000	-1.223184000000
C	-3.805073000000	-0.701890000000	-0.577694000000
H	-4.797515000000	-0.530375000000	-0.975953000000
C	-3.039336000000	0.516193000000	-0.072685000000
C	-1.096200000000	2.362519000000	0.707967000000
H	-0.392031000000	3.121845000000	1.028807000000
C	-2.424878000000	2.661463000000	0.767109000000
H	-2.755831000000	3.633324000000	1.117515000000
C	-3.427911000000	1.727967000000	0.375995000000
H	-4.476455000000	1.983654000000	0.419215000000
H	-4.356745000000	-2.085747000000	1.209856000000
H	-1.767554000000	-2.803136000000	1.015251000000
N	-1.728278000000	0.143852000000	-0.156112000000
B	-0.649184000000	0.990781000000	0.227344000000
C	0.843117000000	0.501944000000	0.138564000000
C	1.228568000000	-0.783208000000	0.523423000000
C	1.831175000000	1.377163000000	-0.314721000000
C	2.549528000000	-1.173484000000	0.448812000000
C	3.146106000000	0.970373000000	-0.394042000000
C	3.525501000000	-0.307486000000	-0.014634000000
H	0.515393000000	-1.496109000000	0.915531000000
F	4.793643000000	-0.689993000000	-0.088106000000
H	1.587658000000	2.386171000000	-0.619580000000

F	2.913456000000	-2.399173000000	0.826739000000
F	4.086628000000	1.803094000000	-0.837654000000

2-B$_{AA}$

***N*$_{imag}$ =** 0

***G*$_{DLPNO-CCSD(T)}$ =** -1189.783212

O	-2.986915000000	-1.960765000000	0.164282000000
C	-3.649711000000	-0.605312000000	1.886454000000
C	-2.368986000000	-0.881032000000	2.076517000000
C	-1.862526000000	-1.308527000000	0.697985000000
H	-0.958900000000	-1.898164000000	0.616044000000
C	-3.895424000000	-0.882272000000	0.400988000000
H	-4.901079000000	-1.108759000000	0.068936000000
C	-3.149903000000	0.225048000000	-0.336930000000
C	-1.229957000000	1.973933000000	-1.369085000000
H	-0.534386000000	2.688551000000	-1.791862000000
C	-2.564601000000	2.194234000000	-1.547238000000
H	-2.908420000000	3.061511000000	-2.100554000000
C	-3.554101000000	1.314860000000	-1.023554000000
H	-4.606613000000	1.507849000000	-1.170623000000
H	-4.367022000000	-0.179685000000	2.568991000000
H	-1.759679000000	-0.750040000000	2.955617000000
N	-1.833340000000	-0.069260000000	-0.133038000000
B	-0.774419000000	0.749063000000	-0.602798000000
C	0.734463000000	0.354379000000	-0.317553000000
C	1.585237000000	1.207819000000	0.368746000000
C	1.296219000000	-0.829545000000	-0.769452000000
C	2.916976000000	0.910976000000	0.604325000000
C	2.623558000000	-1.162472000000	-0.560049000000
C	3.437590000000	-0.283199000000	0.134112000000
F	1.121660000000	2.364417000000	0.843621000000
F	0.545475000000	-1.701446000000	-1.449357000000
F	3.695046000000	1.751240000000	1.273723000000
F	3.120772000000	-2.305093000000	-1.014772000000
F	4.708592000000	-0.583450000000	0.348213000000

1-Al

***N*$_{imag}$ =** 0

***G*$_{DLPNO-CCSD(T)}$ =** -799.329214

C	3.515403000000	-1.161524000000	0.007278000000
C	2.685908000000	1.381660000000	-0.001484000000
H	2.486162000000	2.447526000000	-0.004897000000
C	4.009156000000	1.038744000000	0.002080000000
H	4.767634000000	1.817535000000	0.001364000000
C	4.544361000000	-0.302269000000	0.006926000000
H	5.588741000000	-0.550800000000	0.009625000000
N	2.381149000000	-1.519200000000	0.006073000000
Al	1.330033000000	0.029052000000	-0.000295000000
C	-0.611158000000	-0.017369000000	-0.002300000000
C	-1.309758000000	1.204871000000	-0.003909000000
C	-1.357019000000	-1.211565000000	-0.005793000000
C	-2.698605000000	1.217451000000	-0.007242000000
C	-2.744911000000	-1.162784000000	-0.009195000000
C	-3.434677000000	0.041703000000	-0.007451000000
H	-3.220348000000	2.167923000000	-0.012066000000
H	-3.305769000000	-2.090785000000	-0.015641000000
C	-0.686133000000	-2.561398000000	-0.009022000000
H	-0.049446000000	-2.693710000000	0.866618000000
H	-1.422842000000	-3.363231000000	-0.011567000000

H	-0.048227000000	-2.689315000000	-0.884458000000
C	-0.578400000000	2.525225000000	-0.005251000000
H	-1.276287000000	3.360866000000	-0.005596000000
H	0.061083000000	2.630600000000	0.873701000000
H	0.060654000000	2.629337000000	-0.884668000000
C	-4.936524000000	0.068608000000	0.021547000000
H	-5.326104000000	0.989848000000	-0.410467000000
H	-5.358512000000	-0.773273000000	-0.526967000000
H	-5.301072000000	0.006101000000	1.049240000000

1-Ga

$\boldsymbol{N_{imag}}$ **=** 0

$\boldsymbol{G_{DLPNO\text{-}CCSD(T)}}$ **=** −2480.786040

C	3.314267000000	1.220105000000	0.014489000000
C	2.554822000000	-1.366720000000	-0.007037000000
H	2.358449000000	-2.431403000000	-0.015773000000
C	3.866555000000	-0.976933000000	-0.000921000000
H	4.639171000000	-1.740972000000	-0.004873000000
C	4.359066000000	0.365331000000	0.010362000000
H	5.397861000000	0.637301000000	0.014703000000
N	2.186464000000	1.580210000000	0.013860000000
Ga	1.131914000000	-0.100563000000	-0.001630000000
C	-0.807278000000	0.016838000000	-0.003099000000
C	-1.508547000000	-1.201100000000	-0.002498000000
C	-1.527666000000	1.222064000000	-0.008656000000
C	-2.896888000000	-1.197927000000	-0.004946000000
C	-2.916751000000	1.183159000000	-0.011211000000
C	-3.619377000000	-0.013393000000	-0.006633000000
H	-3.428475000000	-2.142782000000	-0.007963000000
H	-3.467479000000	2.116975000000	-0.019205000000
C	-0.843629000000	2.562654000000	-0.014444000000
H	-0.195807000000	2.676833000000	-0.883705000000
H	-1.574372000000	3.369639000000	-0.027249000000
H	-0.211211000000	2.691028000000	0.864155000000
C	-0.784934000000	-2.524775000000	-0.002807000000
H	-1.487726000000	-3.355993000000	-0.000141000000
H	-0.149187000000	-2.633987000000	-0.884275000000
H	-0.144922000000	-2.632108000000	0.875786000000
C	-5.121412000000	-0.025160000000	0.024183000000
H	-5.521396000000	-0.921127000000	-0.449645000000
H	-5.483519000000	-0.007890000000	1.054470000000
H	-5.535327000000	0.845625000000	-0.483478000000

1-In

$\boldsymbol{N_{imag}}$ **=** 0

$\boldsymbol{G_{DLPNO\text{-}CCSD(T)}}$ **=** −746.794562

C	-3.083275000000	1.094298000000	1.032333000000
C	-2.851764000000	-0.975409000000	-0.825690000000
H	-2.887325000000	-1.779541000000	-1.548025000000
C	-4.042460000000	-0.487412000000	-0.352781000000
H	-4.954575000000	-0.942374000000	-0.731316000000
C	-4.251259000000	0.554561000000	0.586089000000
H	-5.222621000000	0.881821000000	0.909438000000
N	-1.938563000000	1.321420000000	1.198656000000
In	-1.038833000000	-0.217888000000	-0.205327000000
C	1.068580000000	-0.059060000000	-0.089541000000
C	1.826417000000	-1.200054000000	0.192796000000
C	1.704987000000	1.171108000000	-0.288250000000
C	3.209367000000	-1.094933000000	0.278400000000

C	3.089624000000	1.238276000000	-0.195494000000
C	3.857957000000	0.117656000000	0.090329000000
H	3.795608000000	-1.980797000000	0.495024000000
H	3.583053000000	2.190748000000	-0.352945000000
C	0.919331000000	2.417448000000	-0.599438000000
H	0.234796000000	2.663544000000	0.213596000000
H	1.575693000000	3.271003000000	-0.760513000000
H	0.320762000000	2.291372000000	-1.505695000000
C	1.170203000000	-2.539477000000	0.412615000000
H	1.907695000000	-3.324610000000	0.570771000000
H	0.519497000000	-2.522912000000	1.290475000000
H	0.562079000000	-2.833667000000	-0.446601000000
C	5.351576000000	0.222221000000	0.218406000000
H	5.755120000000	0.977689000000	-0.455349000000
H	5.630310000000	0.506854000000	1.235252000000
H	5.836967000000	-0.727899000000	-0.002297000000

TS-Al

$\boldsymbol{N_{imag}}$ **=** 1, -281 cm^{-1}
$\boldsymbol{G_{DLPNO\text{-}CCSD(T)}}$ **=** -1028.884162

O	-3.487413000000	-1.513739000000	-1.084234000000
C	-4.119373000000	-1.045351000000	1.027172000000
C	-3.183881000000	-2.047244000000	1.071162000000
C	-2.733282000000	-2.189231000000	-0.241303000000
H	-2.014153000000	-2.868963000000	-0.665815000000
C	-4.112862000000	-0.556095000000	-0.309402000000
H	-4.922421000000	-0.045528000000	-0.806443000000
C	-2.725173000000	0.604143000000	-0.146262000000
C	-0.800624000000	2.761773000000	0.009366000000
H	-0.219822000000	3.675385000000	0.089939000000
C	-2.157359000000	2.935589000000	-0.018657000000
H	-2.559704000000	3.946683000000	0.023862000000
C	-3.145218000000	1.907881000000	-0.091146000000
H	-4.194860000000	2.156665000000	-0.130720000000
H	-4.631094000000	-0.576539000000	1.850584000000
H	-2.785376000000	-2.554444000000	1.932093000000
N	-1.631447000000	-0.006831000000	-0.121112000000
Al	-0.117179000000	0.980967000000	-0.052067000000
C	1.694768000000	0.235638000000	-0.027219000000
C	2.799898000000	1.103902000000	0.060859000000
C	1.952799000000	-1.146269000000	-0.081983000000
C	4.093761000000	0.597737000000	0.092383000000
C	3.257277000000	-1.623531000000	-0.047924000000
C	4.343919000000	-0.765389000000	0.037262000000
H	4.929720000000	1.285034000000	0.164048000000
H	3.431942000000	-2.693317000000	-0.087606000000
C	0.828861000000	-2.146348000000	-0.182091000000
H	0.246971000000	-1.987340000000	-1.091872000000
H	1.210508000000	-3.166839000000	-0.195444000000
H	0.136926000000	-2.048967000000	0.656023000000
C	2.616300000000	2.600176000000	0.128250000000
H	3.576629000000	3.112539000000	0.165082000000
H	2.070723000000	2.975770000000	-0.739282000000
H	2.048651000000	2.893510000000	1.013497000000
C	5.750536000000	-1.294489000000	0.039796000000
H	6.416182000000	-0.648460000000	0.611991000000
H	5.797114000000	-2.296810000000	0.465160000000
H	6.142753000000	-1.350578000000	-0.978144000000

TS-Ga

N

$\boldsymbol{N_{imag}}$ **=** 1, -285 cm^{-1}

$\boldsymbol{G_{DLPNO\text{-}CCSD(T)}}$ **=** -2710.339155

O	-3.490677000000	-1.633946000000	-1.074608000000
C	-4.105458000000	-1.184284000000	1.046112000000
C	-3.163711000000	-2.180140000000	1.074288000000
C	-2.721462000000	-2.307948000000	-0.243277000000
H	-2.003559000000	-2.981640000000	-0.679146000000
C	-4.110994000000	-0.683612000000	-0.286736000000
H	-4.927222000000	-0.172496000000	-0.772545000000
C	-2.720943000000	0.482240000000	-0.139049000000
C	-0.818166000000	2.671555000000	0.016978000000
H	-0.227532000000	3.576666000000	0.098580000000
C	-2.177161000000	2.821886000000	-0.010868000000
H	-2.585994000000	3.829683000000	0.032839000000
C	-3.148578000000	1.788673000000	-0.083578000000
H	-4.200306000000	2.028816000000	-0.118156000000
H	-4.612535000000	-0.724961000000	1.877720000000
H	-2.755041000000	-2.691750000000	1.927850000000
N	-1.634031000000	-0.130432000000	-0.120778000000
Ga	-0.073684000000	0.918495000000	-0.042172000000
C	1.721056000000	0.133537000000	-0.023147000000
C	2.826124000000	0.996506000000	0.068537000000
C	1.955545000000	-1.248503000000	-0.095505000000
C	4.115085000000	0.477184000000	0.089284000000
C	3.256849000000	-1.735175000000	-0.072224000000
C	4.351425000000	-0.887852000000	0.022750000000
H	4.957658000000	1.156552000000	0.156620000000
H	3.421939000000	-2.805393000000	-0.133078000000
C	0.820289000000	-2.231341000000	-0.207036000000
H	0.231803000000	-2.044450000000	-1.106798000000
H	1.190657000000	-3.255302000000	-0.243879000000
H	0.137163000000	-2.139861000000	0.638617000000
C	2.649405000000	2.492109000000	0.143342000000
H	3.611584000000	2.998883000000	0.199248000000
H	2.119811000000	2.875117000000	-0.731031000000
H	2.068574000000	2.781071000000	1.021478000000
C	5.749953000000	-1.435265000000	0.079519000000
H	6.470783000000	-0.725013000000	-0.324846000000
H	6.039616000000	-1.646310000000	1.111374000000
H	5.834880000000	-2.365790000000	-0.481539000000

TS-In

$\boldsymbol{N_{imag}}$ **=** 1, -367 cm^{-1}

$\boldsymbol{G_{DLPNO\text{-}CCSD(T)}}$ **=** -976.335532

O	-3.521928000000	-1.842453000000	-1.072419000000
C	-4.158980000000	-1.461747000000	1.056595000000
C	-3.132959000000	-2.365986000000	1.073696000000
C	-2.665867000000	-2.421817000000	-0.246885000000
H	-1.902320000000	-3.038783000000	-0.688988000000
C	-4.199841000000	-0.942891000000	-0.272315000000
H	-5.053193000000	-0.484751000000	-0.747811000000
C	-2.861033000000	0.294046000000	-0.139027000000
C	-1.125233000000	2.721497000000	0.014326000000
H	-0.645910000000	3.689724000000	0.093611000000
C	-2.491488000000	2.697808000000	-0.017510000000

H	-3.005345000000	3.657356000000	0.023064000000
C	-3.361314000000	1.579855000000	-0.088780000000
H	-4.428270000000	1.741624000000	-0.120073000000
H	-4.706350000000	-1.060402000000	1.892647000000
H	-2.679471000000	-2.848743000000	1.921719000000
N	-1.759252000000	-0.286327000000	-0.124600000000
In	-0.058580000000	0.947773000000	-0.034071000000
C	1.878871000000	0.058512000000	-0.015334000000
C	2.992064000000	0.904931000000	0.089213000000
C	2.080068000000	-1.325083000000	-0.092180000000
C	4.271214000000	0.362750000000	0.114242000000
C	3.373304000000	-1.834929000000	-0.064265000000
C	4.481995000000	-1.006872000000	0.036200000000
H	5.125878000000	1.024829000000	0.198979000000
H	3.519486000000	-2.908022000000	-0.120900000000
C	0.920260000000	-2.277922000000	-0.207571000000
H	0.345600000000	-2.082059000000	-1.114927000000
H	1.260117000000	-3.312550000000	-0.234760000000
H	0.230702000000	-2.163315000000	0.630601000000
C	2.829900000000	2.401608000000	0.179335000000
H	3.794064000000	2.902613000000	0.250392000000
H	2.315461000000	2.799947000000	-0.698295000000
H	2.245057000000	2.687240000000	1.056899000000
C	5.874010000000	-1.573704000000	0.031732000000
H	5.890343000000	-2.589797000000	0.425076000000
H	6.272957000000	-1.607526000000	-0.984482000000
H	6.551959000000	-0.964698000000	0.629469000000

2-Al

N_{imag} = 0

$G_{DLPNO\text{-}CCSD(T)}$ = −1028.933649

C	3.515403000000	-1.161524000000	0.007278000000
C	2.685908000000	1.381660000000	-0.001484000000
H	2.486162000000	2.447526000000	-0.004897000000
C	4.009156000000	1.038744000000	0.002080000000
H	4.767634000000	1.817535000000	0.001364000000
C	4.544361000000	-0.302269000000	0.006926000000
H	5.588741000000	-0.550800000000	0.009625000000
N	2.381149000000	-1.519200000000	0.006073000000
Al	1.330033000000	0.029052000000	-0.000295000000
C	-0.611158000000	-0.017369000000	-0.002300000000
C	-1.309758000000	1.204871000000	-0.003909000000
C	-1.357019000000	-1.211565000000	-0.005793000000
C	-2.698605000000	1.217451000000	-0.007242000000
C	-2.744911000000	-1.162784000000	-0.009195000000
C	-3.434677000000	0.041703000000	-0.007451000000
H	-3.220348000000	2.167923000000	-0.012066000000
H	-3.305769000000	-2.090785000000	-0.015641000000
C	-0.686133000000	-2.561398000000	-0.009022000000
H	-0.049446000000	-2.693710000000	0.866618000000
H	-1.422842000000	-3.363231000000	-0.011567000000
H	-0.048227000000	-2.689315000000	-0.884458000000
C	-0.578400000000	2.525225000000	-0.005251000000
H	-1.276287000000	3.360866000000	-0.005596000000
H	0.061083000000	2.630600000000	0.873701000000
H	0.060654000000	2.629337000000	-0.884668000000
C	-4.936524000000	0.068608000000	0.021547000000
H	-5.326104000000	0.989848000000	-0.410467000000
H	-5.358512000000	-0.773273000000	-0.526967000000
H	-5.301072000000	0.006101000000	1.049240000000

2-Ga

N_{imag} = 0

$G_{DLPNO-CCSD(T)}$ = −2710.387384

C	3.314267000000	1.220105000000	0.014489000000
C	2.554822000000	-1.366720000000	-0.007037000000
H	2.358449000000	-2.431403000000	-0.015773000000
C	3.866555000000	-0.976933000000	-0.000921000000
H	4.639171000000	-1.740972000000	-0.004873000000
C	4.359066000000	0.365331000000	0.010362000000
H	5.397861000000	0.637301000000	0.014703000000
N	2.186464000000	1.580210000000	0.013860000000
Ga	1.131914000000	-0.100563000000	-0.001630000000
C	-0.807278000000	0.016838000000	-0.003099000000
C	-1.508547000000	-1.201100000000	-0.002498000000
C	-1.527666000000	1.222064000000	-0.008656000000
C	-2.896888000000	-1.197927000000	-0.004946000000
C	-2.916751000000	1.183159000000	-0.011211000000
C	-3.619377000000	-0.013393000000	-0.006633000000
H	-3.428475000000	-2.142782000000	-0.007963000000
H	-3.467479000000	2.116975000000	-0.019205000000
C	-0.843629000000	2.562654000000	-0.014444000000
H	-0.195807000000	2.676833000000	-0.883705000000
H	-1.574372000000	3.369639000000	-0.027249000000
H	-0.211211000000	2.691028000000	0.864155000000
C	-0.784934000000	-2.524775000000	-0.002807000000
H	-1.487726000000	-3.355993000000	-0.000141000000
H	-0.149187000000	-2.633987000000	-0.884275000000
H	-0.144922000000	-2.632108000000	0.875786000000
C	-5.121412000000	-0.025160000000	0.024183000000
H	-5.521396000000	-0.921127000000	-0.449645000000
H	-5.483519000000	-0.007890000000	1.054470000000
H	-5.535327000000	0.845625000000	-0.483478000000

2-In

N_{imag} = 0

$G_{DLPNO-CCSD(T)}$ = −976.380807

C	-3.083275000000	1.094298000000	1.032333000000
C	-2.851764000000	-0.975409000000	-0.825690000000
H	-2.887325000000	-1.779541000000	-1.548025000000
C	-4.042460000000	-0.487412000000	-0.352781000000
H	-4.954575000000	-0.942374000000	-0.731316000000
C	-4.251259000000	0.554561000000	0.586089000000
H	-5.222621000000	0.881821000000	0.909438000000
N	-1.938563000000	1.321420000000	1.198656000000
In	-1.038833000000	-0.217888000000	-0.205327000000
C	1.068580000000	-0.059060000000	-0.089541000000
C	1.826417000000	-1.200054000000	0.192796000000
C	1.704987000000	1.171108000000	-0.288250000000
C	3.209367000000	-1.094933000000	0.278400000000
C	3.089624000000	1.238276000000	-0.195494000000
C	3.857957000000	0.117656000000	0.090329000000
H	3.795608000000	-1.980797000000	0.495024000000
H	3.583053000000	2.190748000000	-0.352945000000
C	0.919331000000	2.417448000000	-0.599438000000
H	0.234796000000	2.663544000000	0.213596000000
H	1.575693000000	3.271003000000	-0.760513000000
H	0.320762000000	2.291372000000	-1.505695000000
C	1.170203000000	-2.539477000000	0.412615000000

H	1.907695000000	-3.324610000000	0.570771000000
H	0.519497000000	-2.522912000000	1.290475000000
H	0.562079000000	-2.833667000000	-0.446601000000
C	5.351576000000	0.222221000000	0.218406000000
H	5.755120000000	0.977689000000	-0.455349000000
H	5.630310000000	0.506854000000	1.235252000000
H	5.836967000000	-0.727899000000	-0.002297000000
